# Precision Targeting of Tumor Macrophages with a CD206 Binding Peptide

**DOI:** 10.1038/s41598-017-14709-x

**Published:** 2017-11-07

**Authors:** Pablo Scodeller, Lorena Simón-Gracia, Sergei Kopanchuk, Allan Tobi, Kalle Kilk, Pille Säälik, Kaarel Kurm, Mario Leonardo Squadrito, Venkata Ramana Kotamraju, Ago Rinken, Michele De Palma, Erkki Ruoslahti, Tambet Teesalu

**Affiliations:** 1 Laboratory of Cancer Biology, Institute of Biomedicine and Translational Medicine, University of Tartu, Ravila 14B, Tartu, 50411 Estonia; 2 Cancer Research Center, Sanford Burnham Prebys Medical Discovery Institute, 10901 N, Torrey Pines Road, La Jolla, 92097 California, USA; 3 Institute of Chemistry, University of Tartu, Ravila 14, Tartu, 50411 Estonia; 4 Department of Biochemistry, Institute of Biomedicine and Translational Medicine, University of Tartu, Ravila 14B, Tartu, 50411 Estonia; 5 School of Life Sciences, Ecole Polytechnique Fédérale de Lausanne (EPFL), Swiss Federal Institute of Technology, Lausanne, CH-1015 Lausanne, Switzerland; 6 Center for Nanomedicine and Department of Cell, Molecular and Developmental Biology, University of California, Santa Barbara Santa Barbara, 93106 California, USA

## Abstract

Tumor-associated macrophages (TAMs) expressing the multi-ligand endocytic receptor mannose receptor (CD206/MRC1) contribute to tumor immunosuppression, angiogenesis, metastasis, and relapse. Here, we describe a peptide that selectively targets **M**RC1-**e**xpressing TA**M**s (MEMs). We performed *in vivo* peptide phage display screens in mice bearing 4T1 metastatic breast tumors to identify peptides that target peritoneal macrophages. Deep sequencing of the peptide-encoding inserts in the selected phage pool revealed enrichment of the peptide CSPGAKVRC (codenamed “UNO”). Intravenously injected FAM-labeled UNO (FAM-UNO) homed to tumor and sentinel lymph node MEMs in different cancer models: 4T1 and MCF-7 breast carcinoma, B16F10 melanoma, WT-GBM glioma and MKN45-P gastric carcinoma. Fluorescence anisotropy assay showed that FAM-UNO interacts with recombinant CD206 when subjected to reducing conditions. Interestingly, the GSPGAK motif is present in all CD206-binding collagens. FAM-UNO was able to transport drug-loaded nanoparticles into MEMs, whereas particles without the peptide were not taken up by MEMs. In *ex vivo* organ imaging, FAM-UNO showed significantly higher accumulation in sentinel lymph nodes than a control peptide. This study suggests applications for UNO peptide in diagnostic imaging and therapeutic targeting of MEMs in solid tumors.

## Introduction

Tumor-associated macrophages displaying a M2-like phenotype (M2 TAMs) play major roles in progression of solid tumors, including epithelial and mesenchymal tumors, glia-derived tumors, and melanoma^1^. M2-like TAMs promote tumor growth and progression by stimulating tumor cell proliferation^2–5^, and by secreting factors that promote angiogenesis, such as VEGF-A^6^. M2 TAMs also induce transient openings in tumor neovessels that allow malignant cells to enter the bloodstream, promoting metastatic dissemination of solid tumors^1,7^. M2-like TAMs increase in number after chemotherapy and contribute to tumor relapse^2,6^. They also limit the efficacy of chemotherapies^8^ and support immunosuppressive microenvironment in tumors^9^. The immunosuppressive effect is partly mediated through expression of ligands for the inhibitor receptors PD-1 (programmed cell death protein 1) and cytotoxic T-Lymphocyte Antigen-4 (CTLA-4)^5^. The M2 differentiation state is supported in part by the exposure to Th2 cytokines, such as IL-4 and IL-13, which results in (1) upregulation of the anti-inflammatory cytokine, IL-10, (2) decreased expression of pro-inflammatory cytokines, (3) amplification of metabolic pathways that suppress adaptive immune responses, and (4) upregulation of cell-surface scavenger receptors such as the mannose receptor (MRC1/CD206) and the hemoglobin/haptoglobin scavenger receptor (CD163)^10^. M2-like TAMs are derived from circulating monocytes that may already express M2-associated markers (such as CD206), which are further upregulated upon extravasation of the cells at the tumor site and by exposure to factors in the perivascular tumor microenvironment^11^.

The appreciation of the central role of M2-like TAMs in tumorigenesis and resistance to therapies has inspired multiple studies aimed to eliminate or reprogram TAMs. We have previously shown that activated TAMs overexpress cell surface p32 protein, a molecule that can be targeted by LyP-1 peptide^12^, its higher-affinity version TT1^13^, and a low-molecular-weight peptidomimetic compound^14^. Remarkably, treatment of tumor mice with the LyP-1 peptide or LyP-1-targeted clodronate nanoparticles caused decrease in TAMs in tumor models, resulting in partial tumor growth inhibition^15,16^. However, cell surface p32 is expressed on activated TAMs, and on other types of cells in tumors, and does not allow specific targeting of M2-skewed macrophages (Säälik *et al*., unpublished). The Manocept™ family of multi-mannose analogue diagnostic imaging compounds targets the lectin domain of CD206. A ^99m^Tc-labeled version of Manocept™, γ-Tilmanocept^10^, is FDA approved for imaging of lymph nodes that drain from a primary tumor and have the highest probability of harboring cancer cells. However, mannose and its analogues are not specific for CD206: they also bind other mannose receptors, such as CD209 expressed in the skin and intestinal and genital mucosa^17,18^. In addition, a nanobody that recognizes CD206 has been developed and its ^99m^Tc and ^18^F-labeled versions have been used for PET imaging of MEMs in mice^19,20^. However, it is not known if the nanobody is internalized by the CD206-positive cells. Recently, a 10-mer peptide, RP-182, was reported to bind CD206^21^. RP-182 (sequence: KFRKAFKRFF) is composed of alternating hydrophobic and hydrophilic amino acids, and is not specific to CD206, as it also binds RelB, SIRP-a, CD47 and TGM2. Finally, other groups have identified peptides that appear to target TAMs^22^, however, the receptors for these peptides are unknown. Here, we identify and characterize a peptide codenamed “UNO” that targets CD206 on MEMs across a spectrum of solid tumors of different types.

## Results

### CSPGAKVRC peptide is enriched in phage display screens on peritoneal macrophages in breast cancer mice

**Figure 1:**
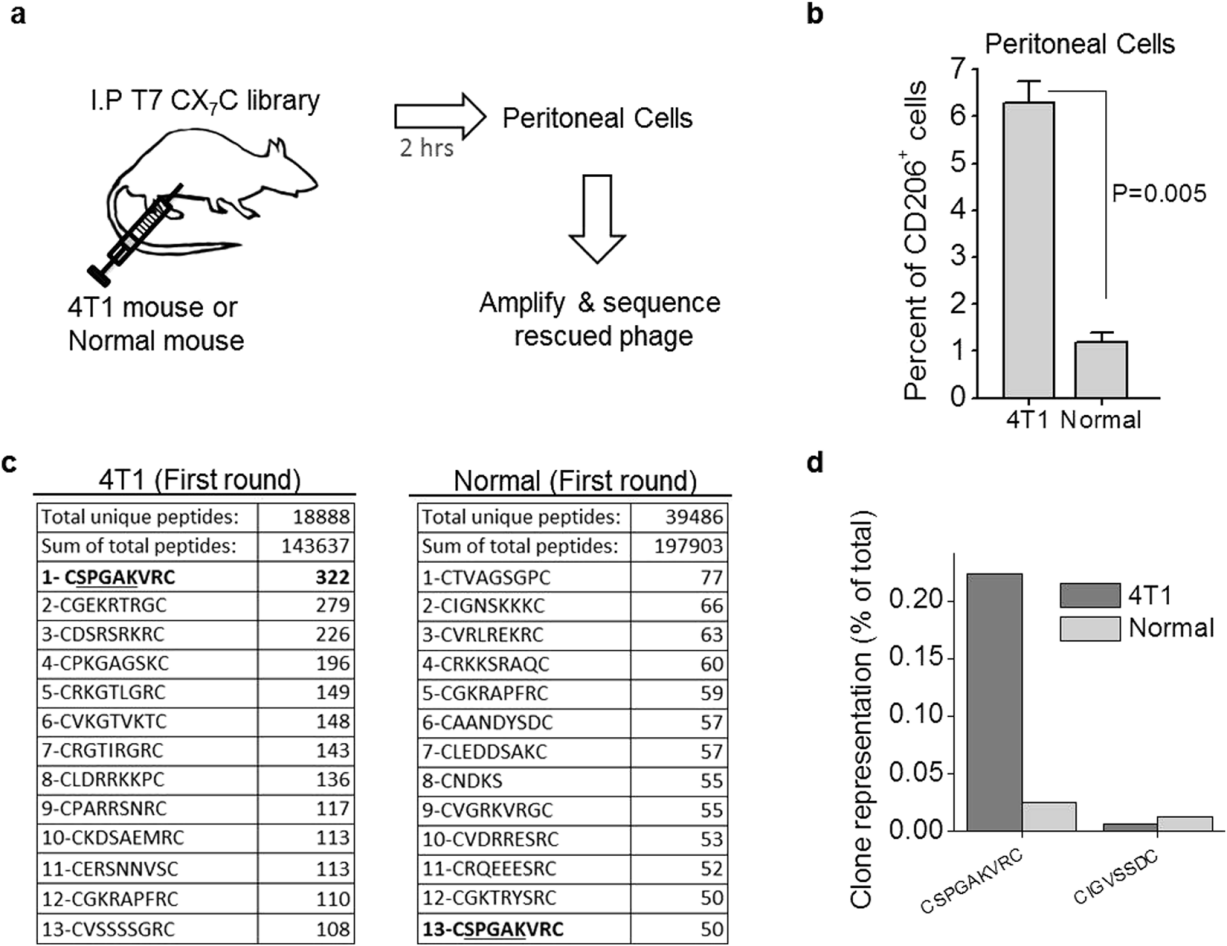
Identification of CSPGAKVRC (“UNO”) in breast cancer mice. (**a**) Naïve phage library was injected intraperitoneally in 4T1 tumor-bearing mice and age-matched normal mice, and allowed to circulate for 2 h. Peritoneal cells were collected, the accompanying phages were rescued and the peptide-encoding segment of phage DNA was sequenced. (**b**) Higher number of CD206^+^ cells were seen in the 4T1 mice than normal mice. Peritoneal cells were extracted from the mice, seeded on coverslips, allowed to attach for 2 h, fixed, permeabilized, and stained for CD206. CD206^+^ cells were counted from 6 different confocal images, 2 from each mouse. (**c**) Highly repeated sequences obtained from the first round of the biopanning experiment are shown schematically in panel A. (**d**) Frequency of phage clones encoding UNO or a randomly picked peptide (CIGVSSDC) divided by the total number of sequences) in the 4T1 tumor-bearing and normal mice.

*In vivo* phage display was carried out on orthotopic breast cancer bearing mice as outlined in Fig. 1a. Compared to peritoneal cells of healthy mice, CD206^+^ macrophages were ~5-fold overrepresented in Balb/c mice bearing metastatic syngeneic 4T1 breast tumors (Fig. 1b). We hypothesized that a peptide that binds to peritoneal macrophages in tumor-bearing mice may also target M2-like TAMs. Mice with 4T1 tumors were injected intraperitoneally (i.p.) with a phage library expressing 9-amino acid cyclic CX7C peptides. After 2 h, peritoneal cells were collected, bound phages were rescued by amplification, and the resulting phage pool was subjected to high-throughput sequencing of the peptide-encoding segment of the phage genome. Among the peptide sequences, CSPGAKVRC was the most highly represented non-truncated peptide (the NNK codons we used to encode the library allow the presence of one stop codon, which results in some truncated peptides). Whereas most peptides showed similar representation in peritoneal cells from the 4T1 tumor-bearing and healthy mice, the CSPGAKVRC-displaying phage was overrepresented in the peritoneal cells from 4T1 tumor-bearing mice (Fig. 1c,d). Interestingly, the second most abundant phage clone overrepresented in the cancer mice was CGEKRTRGC (Fig. 1c), a clone that was previously identified^23^ together with LyP-1 (CGNKRTRGC), a peptide known to target the p32 protein overexpressed on the surface of TAMs and tumor lymphatic endothelial cells^12^. Interestingly, the GSPGAK motif is present in physiological ligands of CD206, a marker of M2-skewed macrophages (Table 1 in Supplementary information) We used the CSPGAKVRC peptide (codenamed “UNO”) for subsequent studies.

### Systemic FAM-UNO targets CD206^+^/TIE2^+^ macrophages in breast cancer models

**Figure 2:**
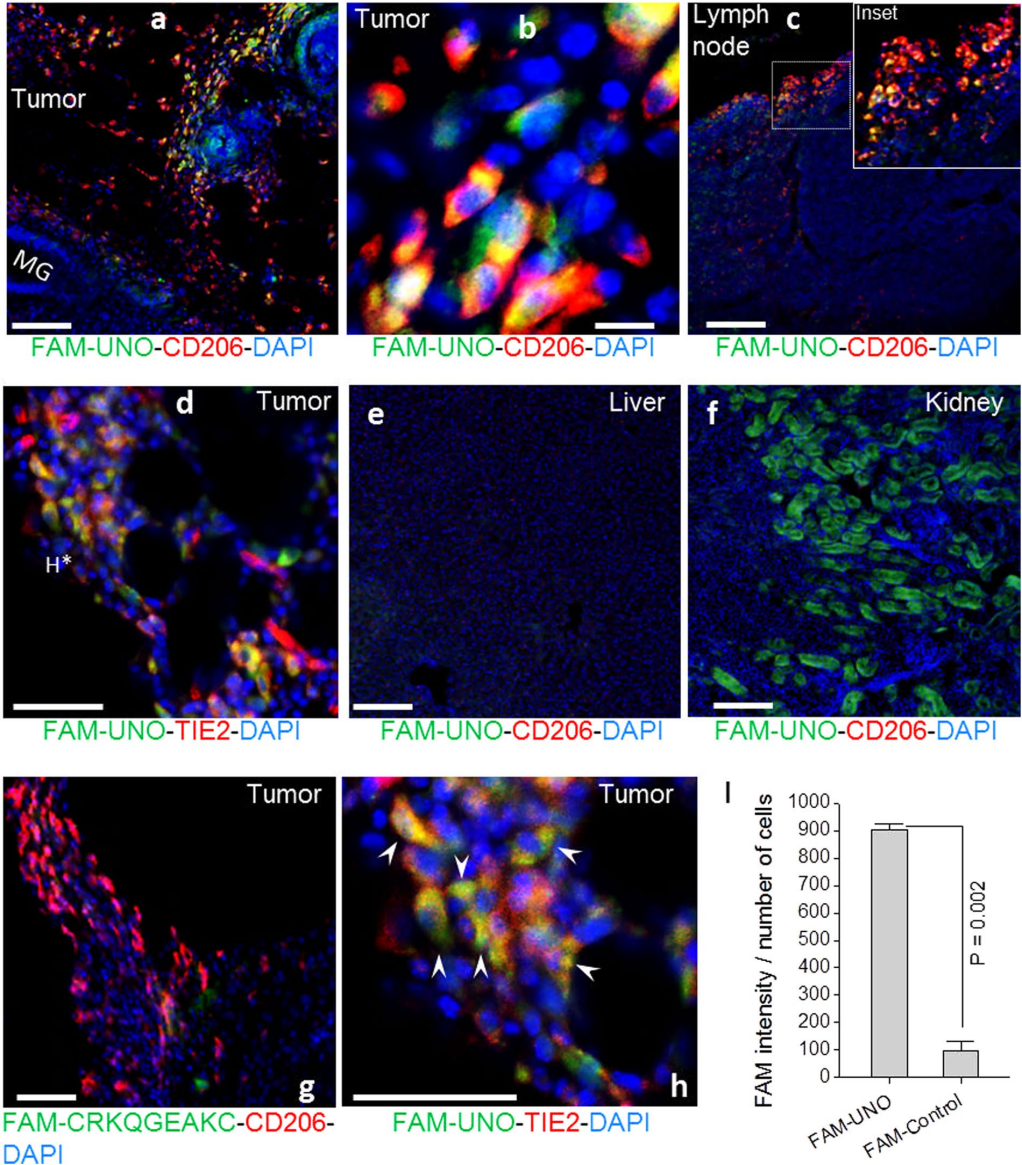
FAM-UNO accumulates in CD206^+^, TIE2^+^ macrophages in breast tumors and lymph nodes. Thirty nmoles of FAM-UNO or FAM-CRKQGEAKC control peptide were injected intravenously into 4T1 tumor-bearing mice and allowed to circulate for 2 h. Mice were then sacrificed and tumor and tissues were analyzed by immunofluorescence using rabbit anti-FAM (green) and rat anti-CD206 or rat anti-TIE2 (red) antibodies, and counterstained with DAPI. All images were taken under the same imaging conditions. FAM-UNO accumulated in macrophages within tumors and lymph nodes positive for CD206 staining (**a**–**c**) and TIE2 (**d**, and **h**: Blow up of **d**). FAM-UNO showed very low accumulation in the liver (**e**), but signal was seen in the kidneys (**f**), which is the normal excretion route for peptides. No signal or only traces of FAM-UNO were observed in the spleen, heart and lungs in images taken under the same conditions (shown in Fig. S4). To ascertain that anti-CD206 is capable of detecting CD206 in the liver, an image was acquired with higher gain from an uninjected animal (Fig. S19). The charge-matched control peptide, CRKQGEAKC, did not give any signal in CD206^+^ macrophages or elsewhere in the tumor (**g** and **i**). Green: FAM-peptide; Red: CD206 or TIE2, Blue: DAPI. Representative fields from multiple sections prepared from at least 3 tumors (n ≥ 3 mice) are shown. The graph in panel I shows mean + SEM of FAM signal quantified as described in materials and methods. Scale bars: 100 µm for a, b, c, e, f, g and 50 µm for d and h.

To assess *in vivo* targeting potential of synthetic UNO peptide, we studied the biodistribution of intravenously (i.v.) injected, fluorescently labeled UNO (FAM-UNO), in orthotopic 4T1 tumor-bearing mice and compared it with a FAM-labeled control peptide. FAM-UNO accumulated in MEMs in tumors and in sentinel lymph nodes (Fig. 2a–c). High-resolution confocal microscopy, using low optical slice thickness, suggested that FAM-UNO was internalized in MEMs (Supplementary material: Fig. S1 and Video 1). The MEMs containing FAM-UNO were also positive for angiopoietin receptor TIE2^+^ (Fig. 2d,h). The MEMs were highly abundant in the tumors and the sentinel lymph nodes (Fig. S2), and their distribution correlated with the homing pattern of FAM-UNO. In tumors, 96% of cells positive for FAM-UNO were also positive for CD206. The signal from FAM-UNO remained readily detectable in the MEMs for up to 12 h after peptide administration (Fig. S3). As a specificity control, we injected 4T1 tumor-bearing mice with a control peptide of the same charge and molecular weight as FAM-UNO, FAM-CRKQGEAKC (FAM-control), and saw no tumor homing (Fig. 2g). The accumulation of FAM-UNO was low in the liver (Fig. 2e), heart, lungs and spleen (Fig. S4). At the 2 h time point, FAM-fluorescence was observed in the kidneys, which are the normal excretion route for peptides (Fig. 2f). We also studied the biodistribution of i.v.-injected FAM-UNO in an orthotopic estrogen receptor (ER)-positive xenograft model of breast cancer, MCF-7. FAM-UNO also selectively accumulated in MEMs in this tumor model (Fig. S5A). The accumulation of FAM-UNO in some CD206^−^ regions (arrowhead in blow-up of Fig. S5A) did not correlate with the presence of HLA ABC^+^ cells (Fig. S5B), suggesting that FAM-UNO did not target the tumor cells of human origin. Instead, the accumulation of FAM-UNO in such regions was likely due to entrapment of extravasated peptide, as the vessels in these tumors appeared to be leaky as suggested by immunostaining for mouse endogenous IgG (Fig. S5C).

### FAM-UNO targets MEMs across a spectrum of solid tumors of different origins

**Figure 3:**
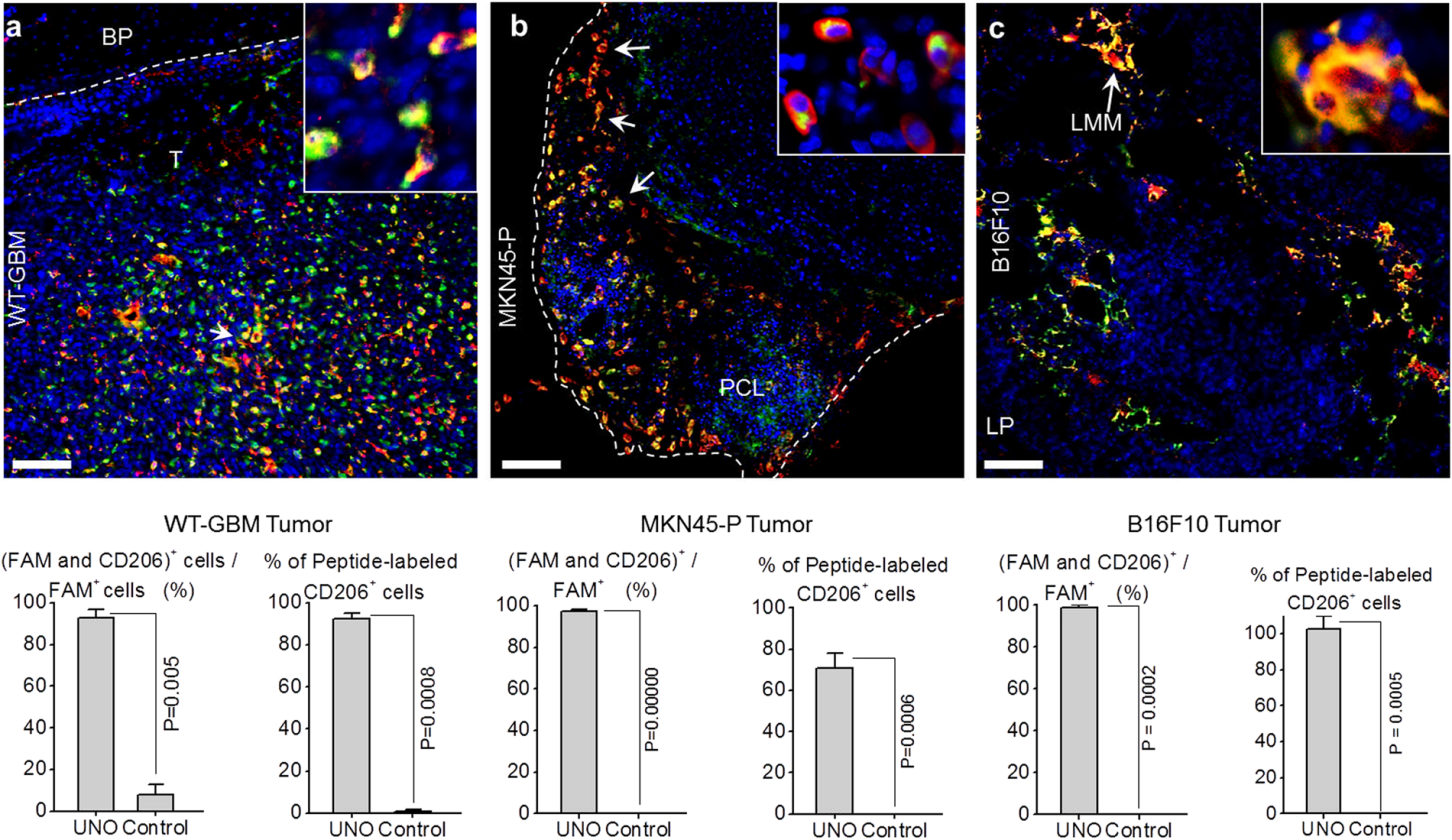
FAM-UNO accumulates in CD206^+^ macrophages in glioblastoma, gastric carcinoma, and melanoma. Thirty nmoles of FAM-UNO or FAM-control peptide were injected intravenously into mice and allowed to circulate for 2 h. Mice were then sacrificed and tumor and tissues were analyzed by immunofluorescence using rabbit anti-FAM (green) and rat anti-CD206 (red) antibodies, and counterstained with DAPI. (**a**) Homing to glioblastoma (WT-GBM). Signal was seen exclusively within the tumor (T) and not in the brain parenchyma (BP). (**b**) Homing to a peritoneal carcinomatosis lesion (PCL) induced by i.p. inoculation of gastric carcinoma cell line MKN4-5P. (**c**) Homing to experimental melanoma metastases in the lungs. The metastases were induced by i.v. inoculation of B16F10 melanoma cells. FAM signal was seen in lung metastases (LMM), and not in noncancerous lung parenchyma (LP). The arrows point to examples of FAM and CD206 colocalization in each panel. The insets show CD206-positive individual cells with internalized FAM-UNO signal. A parallel experiment with the control peptide (FAM-Control) is shown in Fig. S9A–C; images of spleens from mice injected with FAM-UNO are shown in Fig. S13. Blue: DAPI. Representative fields from multiple sections (n ≥ 3) prepared from at least 3 tumors are shown. Scale bar: 50 µm. Images shown are representative fields from multiple sections (≥3) prepared from at least 3 tumors (n ≥ 3 mice). The graphs show mean + SEM of FAM signal quantified as described in Materials and Methods.

We next tested the applicability of UNO for payload targeting using a spectrum of solid tumor models: orthotopic angiogenic glioblastoma (WT-GBM)^24^ generated by stereotactic brain implantation of tumor cells, model of metastatic melanoma (B16F10) generated by intravenous injection of tumor cells, and peritoneal gastric carcinoma (MKN45-P)^25^ generated by intraperitoneal injection of tumor cells. Tumor mice were injected i.v. with FAM-UNO; after 2 h the mice were sacrificed, tissues were collected, and cryosections were analyzed by confocal microscopy. In the WT-GBM tumors, the FAM-UNO peptide accumulated in the tumor areas containing MEMs (Fig. 3a). Intracellular presence of FAM-UNO was also evident in these tumors. The MEMs that internalized FAM-UNO were perivascular (Fig. S6). No signal from FAM-UNO was observed in the normal brain parenchyma (Fig. S7). Even though the blood-brain-barrier is breached in these tumors (Fig. S8), the control peptide did not accumulate in the tumor (Fig. S9A), showing that the tumor homing of UNO is specific and not due to entrapment of the peptide around leaky tumor blood vessels.

Systemic FAM-UNO also accumulated in MEMs in a model of peritoneal carcinomatosis, generated by intraperitoneal inoculation of MKN45-P gastric carcinoma cells (Fig. 3b). In this model, FAM-UNO was detected in the periphery of peritoneal tumor nodules, enriched in MEMs (Fig. S10). Whereas some accumulation of control FAM-peptide in MKN45-P tumors was seen in the regions exhibiting higher vascular leakiness (Figs S9B and S11), no accumulation was detected in CD206^+^ cells (Fig. S9B). Finally, FAM-UNO homing to B16F10 melanoma lung metastases was similar to what we saw with the other tumors (Figs 3c and S9C).

For different tumor models, FAM-UNO and CD206 immunoreactivities showed extensive overlap (Figs S10 and S12). In contrast FAM-UNO did not home to non-malignant control tissues, even in the presence of CD206^+^ macrophages (Fig. S13).

These data suggest that UNO targets MEMs in solid tumors independent of the tumor type and location.

### Linear CSPGAKVRC and CSPGAK bind to CD206

Protein database searches revealed that the GSPGAK motif is present in collagens type I, III and IV (see Table 1 in supplementary information) - all ligands of CD206^26^. In contrast, the motif is absent in type V collagen, which does not bind to CD206^26^. This observation, in combination with the CD206 and FAM-UNO colocalization data, suggested that CD206 might be the target of UNO peptide.

**Figure 4:**
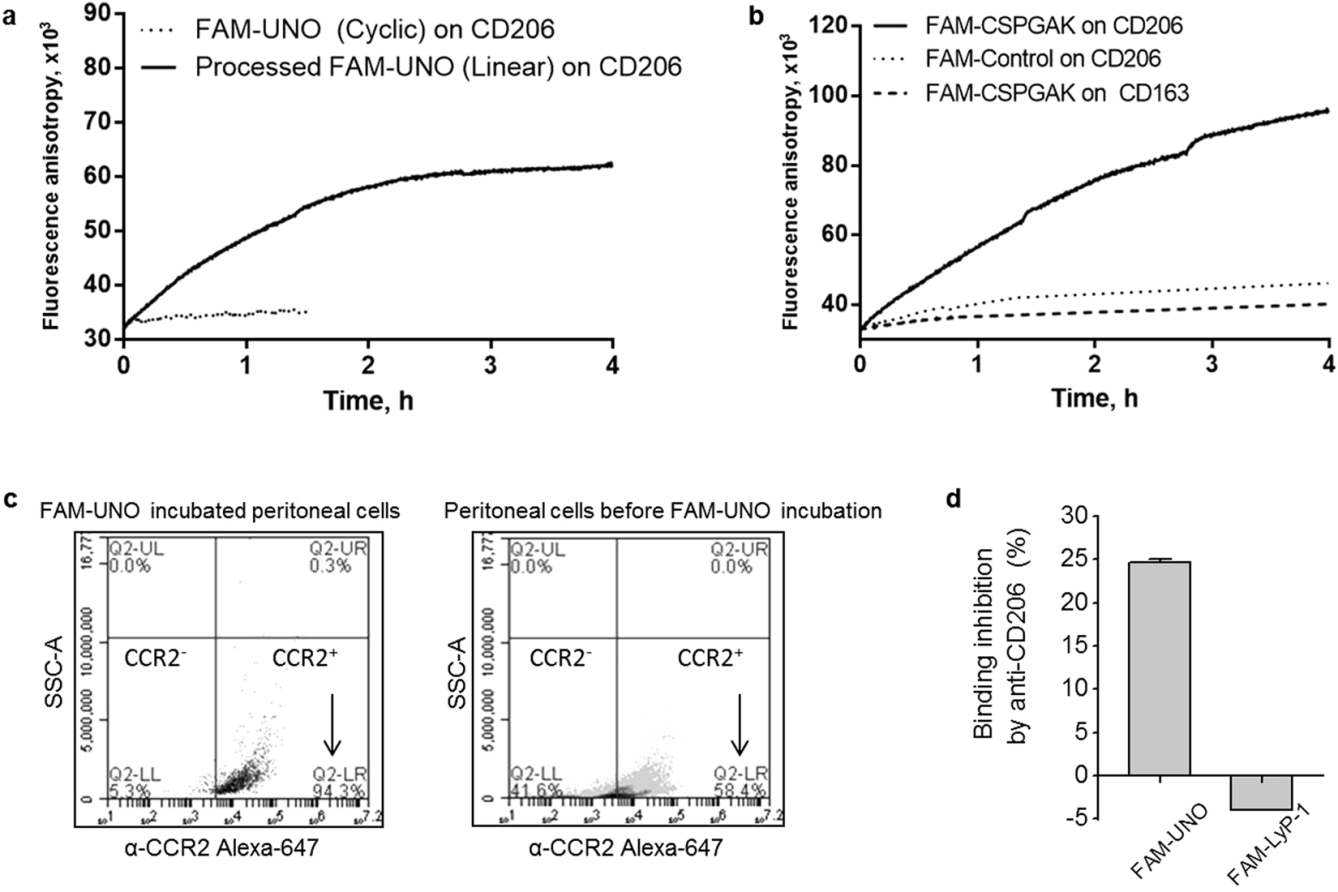
UNO specificity for CD206. (**a**) Change in fluorescence anisotropy of FAM-UNO (dotted line) and FAM-UNO in DTT (solid line) while incubating with mouse recombinant CD206. (**b**) Change in fluorescence anisotropy of FAM-CSPGAK with mouse recombinant CD206 (solid line) or with CD163 (dotted line) and of FAM-CPMTDNE (control) with CD206 (dashed line). (**c**) FAM-UNO binds selectively to CCR2^+^ macrophages collected from the peritoneal cavity of 4T1 tumor-bearing mice, as 94.3% of FAM^+^ cells are CCR2^+^ cells. The analysis was done gating for the FAM^+^ population (left panel). In these 4T1 tumor bearing mice, 58% of peritoneal cells are macrophages, i.e CCR2^+^ cells (right panel). (**d**) FAM-UNO binding to peritoneal cells is inhibited by preincubating with 10 µg/mL of anti-CD206, whereas the preincubation with anti CD206 antibody had no effect on FAM-LyP-1 binding. In panels a and d are shown representative graphs from three independent experiments. In panels c and d are shown results from three independent experiments (n = 3 mice) and bars of panel d represents mean + SEM.

We studied binding of FAM-UNO to mouse recombinant CD206 in cell-free, solution-based, fluorescence anisotropy (FA) assay. Incubation of FAM-UNO with CD206 did not cause a change in the fluorescence anisotropy (Fig. 4a) indicating the absence of interaction. This suggested that if CD206 is the receptor, perhaps the cyclic peptide gets processed *in vivo* to yield a CD206 binder. Liquid-chromatography mass spectrometry (LC-MS) analysis showed that upon incubation of FAM-UNO with the 4T1 tumor lysate, the disulfide bridge was reduced to yield a linear peptide “FAM-LinUNO” (Fig. S14). The levels of glutathione, the cofactor of the glutaredoxin involved in reducing disulfides^27,28^, are known to be elevated in tumors and the reducing capacity in the tumor microenvironment is known to be higher than in healthy tissues^29^. We confirmed that glutathione is present in tumor lysate in the orthotopic 4T1 breast model (Fig. S15). The glutaredoxin system in the tumor microenvironment may contribute to the reduction of the disulfide-bridged FAM-UNO that we observed upon incubation with the tumor lysate. In contrast to FAM-UNO, FAM-LinUNO (obtained by preincubating FAM-UNO with 1,4-dithiothreitol, DTT) did interact with CD206 (Fig. 4a). Since binding to CD206 appeared to require linearity of the peptide, we hypothesized that the linear CSPGAK motif -present in UNO and almost identical to GSPGAK- might contain the minimal CD206-binding motif, and decided to test it in FA assay.

Incubation of FAM-CSPGAK with CD206 caused a clear time-dependent increase of anisotropy, indicating their binding, but there was no change in anisotropy when the peptide was incubated with CD163 (Fig. 4b). The binding of FAM-CSPGAK to CD206 was specific, as incubation of CD206 with a random peptide of similar molecular weight and containing a free cysteine in the same position as FAM-CSPGAK (control peptide FAM-CPMTDNE) did not cause significant changes in anisotropy values (Fig. 4b).

We also excluded the possibility that the control peptide might be dimerized through a disulfide bond (Fig. S16), which would eliminate the free sulfhydryl, possibly accounting for the absence of interaction of FAM-CPMTDNE with CD206.

In line with the interaction of FAM-CSPGAK with recombinant CD206, i.v. administered FAM-CSPGAK accumulated in MEMs in orthotopic 4T1 breast tumor-bearing mice at levels comparable to FAM-UNO (Fig. S17). However, compared to FAM-UNO, the signal from FAM-CSPGAK in non-malignant control tissue that expresses CD206, i.e. liver, was ~7 fold higher.

We next studied the effect of a blocking anti-CD206 antibody on FAM-UNO binding to peritoneal cells isolated from 4T1 tumor mice. Flow cytometry experiments were performed by incubating FAM-peptide with the cells suspended in the peritoneal fluid; this i.p. fluid also contained glutathione (Fig. S18). Flow cytometry analysis showed that 94% of cells gated for the macrophage marker CCR2^+^, known to be coexpressed in mouse peritoneal macrophages with CD206^30^, were positive for FAM-UNO binding. Pre-incubation with the anti-CD206 antibody (validated by immunostaining of liver sections, Fig. S19) reduced the binding by 25% (Fig. 4c). In contrast, anti-CD206 antibody had no effect on the cellular binding of FAM-LyP-1 peptide that targets a different receptor, cell surface p32 (Fig. 4d). No UNO-displaying phages were recovered in a phage library screen on CD206^−^ RAW 267.4 cells (Fig. S20). As an additional indication of homing specificity for FAM-UNO, we observed that the peptide was not recruited to intestinal tissue of healthy mice, known to express another mannose binding C-type lectin, CD209^18^ (Fig. S21A) and that FAM-UNO showed only a moderate overlap with CD209 in sections from 4T1 tumors (Fig. S21B). Therefore UNO recruitment is unlikely to be mediated by a promiscuous binding of FAM-UNO to the family of mannose-binding lectins.

These data established that non-cyclic UNO derivatives interact with CD206 and suggested that conversion of cyclic UNO to linUNO by reducing tumor microenvironment may actuate peptide binding to CD206 *in vivo* for increased tumor selectivity.

### FAM-UNO guides cargo to MEMs and is a potential sentinel lymph node imaging agent

**Figure 5:**
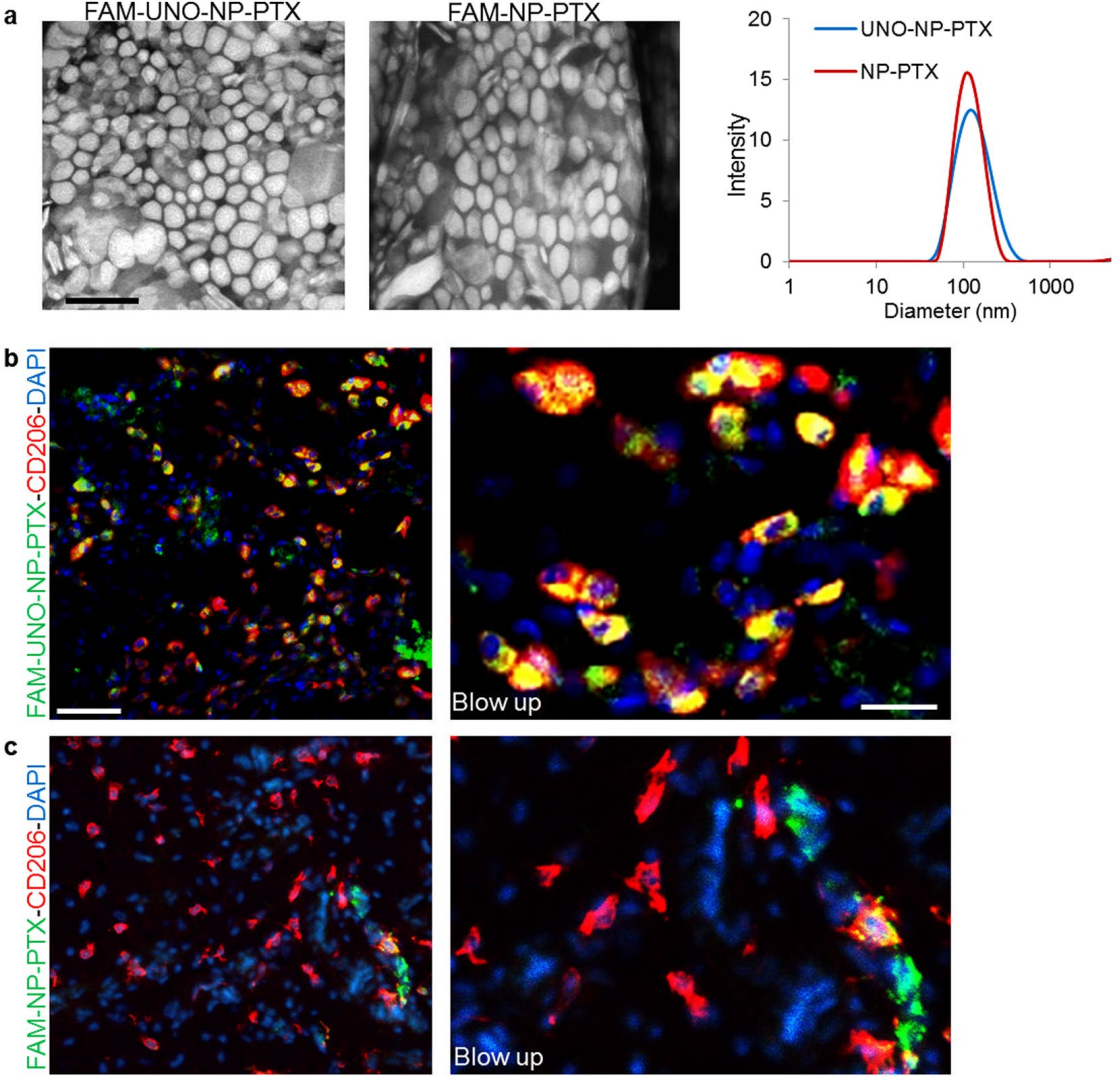
(**a**) Characterization of FAM-UNO derivatized, paclitaxel loaded, polymeric vesicles (“FAM-UNO-NP-PTX”). Transmission electron microscopy images (two panels on the left, scale bar: 200 nm) and Dynamic Light Scattering profile (right panel). Polymeric vesicles are composed of the copolymer polyethylene glycol-polycaprolactone. (**b**,**c**) FAM-UNO guides cargo-loaded nanoparticles inside MEMs. FAM-UNO-NP-PTX were intravenously injected in mice bearing MCF-7 tumors, 21 days after orthotopic inoculation of 5 × 10^6^ cells. The particles were allowed to circulate for 6 h. The mice were then sacrificed, and tumors and tissues were analyzed by immunofluorescence using rabbit anti-FAM (green) and rat anti-CD206 (red) antibodies and counterstained with DAPI (blue). Images shown are representative fields from multiple sections (≥3) prepared from 3 tumors (n = 3 mice).

To assess suitability of UNO for targeting of nanoscale cargo, we coated the peptide on paclitaxel-loaded 120-nm polyethylene glycol-polycaprolactone (PEG-PCL) polymersomes^31^ (Fig. 5a). After i.v. injection in orthotopic MCF-7 breast cancer-bearing mice, the FAM-UNO-derivatized polymersomes loaded with paclitaxel (“FAM-UNO-NP-PTX”) accumulated in MEMs (Fig. 5b), whereas the non-peptide control particles did not (“FAM-NP-PTX”, Fig. 5c). To study whether the FAM-UNO particles are stable, we incubated FAM-UNO-NP-PTX with the sera of 4T1 orthotopic breast tumor mice for 6 h at 37 °C, followed by washes and fluorometry at 520 nm (corresponding to FAM). After incubation ~6% of the initial fluorescence was lost (Fig. S22), indicating that the bulk of the peptide remains on the particles and suggesting that the FAM signal observed in the tumors can be attributed to the FAM-UNO-conjugated particles.

**Figure 6:**
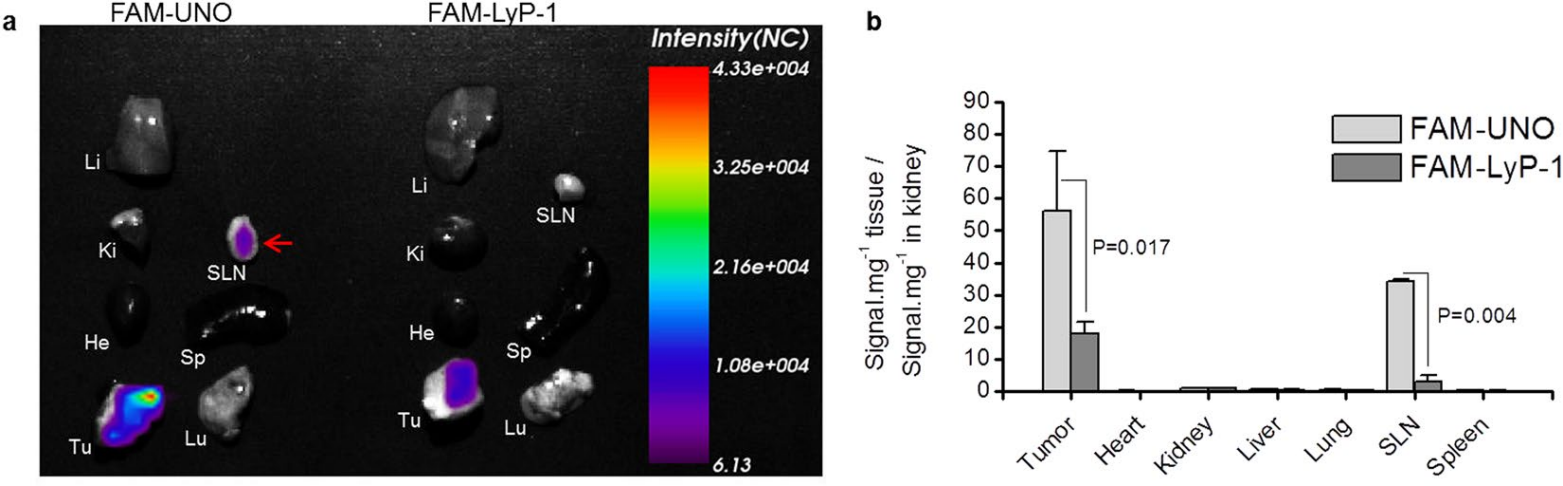
FAM-UNO can be used to image metastasis-draining lymph nodes. (**a**) FAM-UNO and FAM-LyP-1 were injected i.p at doses of 30nmoles in 4T1 tumor-bearing mice. Peptides were allowed to circulate for two h, mice were then sacrificed, and the organs were collected and imaged with the live imaging system MX3 Art Optix in the FITC channel with laser excitation. (**b**) The signal in each organ was quantified, normalized to the tissue weight and the ratio tissue/kidney was graphed in bar graph. LyP-1 is a peptide that targets p32 protein on the surface of activated macrophages^46,47^. Results from n = 3 mice. Bars of panel B represent mean + SEM.

We also evaluated UNO as a guiding module for a contrast agent to image tumor-draining lymph nodes. After administration of FAM-UNO and FAM-LyP-1 to orthotopic 4T1 breast tumor mice, *ex vivo* quantification of the FAM signal showed a significantly higher accumulation of the FAM in the sentinel lymph node for mice injected with FAM-UNO (Fig. 6). FAM-UNO did not home to the lymph nodes of healthy Balb/C mice (Fig. S23). Some tissues such as the kidney absorb the green fluorescence from the FAM probe; the real measure of FAM accumulation in kidney can be based on the immunodetection of the FAM probe on the kidney sections (Fig. 2f). This series of experiments suggested potential applications for UNO-targeted imaging agents for live imaging of tumor-draining lymph nodes.

## Discussion

We have performed *in vivo* phage display on peritoneal cells of tumor bearing mice to identify probes for M2-like TAMs, a cell population recognized to play increasingly important roles in tumor growth and metastasis. We report that a peptide, named UNO, targets MEMs in solid tumors of different origin. We show that UNO is specific for MEMs, and that it effectively delivers payloads, including nanoparticles, into the tumors.

*In vivo* phage display has been successfully used to identify peptides that home to tumors, and to individual cell types in tumors, including macrophages. As this method primarily targets tumor endothelium, it was necessary to remove the endothelial cells in earlier screens for it to yield LyP-1 peptide^23^, shown to recognize tumor lymphatics and activated TAMs^32^. Here, to focus our screening on TAMs, we used peritoneal macrophages from tumor-bearing mice, rather than using tumors as the target.

FAM-UNO accumulated in M2-like TAMs in all 5 different solid tumor models tested, suggesting that the peptide is independent of the origin of the malignancy and location of the tumor. The specificity of UNO is different from the previously known TAM-targeting peptide, LyP-1, which is not selective for MEMs. The selectivity of UNO for MEMs was evident from the extensive colocalization of systemically injected FAM-UNO with CD206, [see quantification of (FAM^+^ and CD206^+^ cells)/FAM^+^ cells on Fig. 3]. The experiments of Fig. 4c showed that FAM-UNO mainly (95%) binds macrophages. These results lead us to conclude that FAM-UNO does not bind macrophages other than MEMs.

Our data indicate that the target molecule (receptor) for UNO in MEMs is CD206. First, our immunofluorescence results show that UNO is highly selective for CD206^+^ cells. Second, linearized FAM-UNO and FAM-CSPGAK bind to recombinant CD206 in fluorescence anisotropy assay. Third, binding of FAM-UNO peptide to peritoneal cells is reduced after pre-incubation with an anti-CD206 blocking antibody. That the antibody inhibition was only partial may be because the anti-CD206 antibody used in this study is monoclonal and the binding epitope on CD206 is likely to be different from the binding epitope for the peptide.

CD206 is a modular protein composed of 3 domains: (1) a mannose-binding lectin domain located closest to the plasma membrane composed of 8 consecutive C-type carbohydrate recognition domains (CRDs)^33–35^; (2) a conserved fibronectin type-II (FNII) domain that interacts with type I, III and IV collagens and their degradation and denaturation products^26,36,37^; (3) a cysteine-rich domain homologous to the ricin B chain that interacts with sulfated glycans^34^. The sharing of the UNO sequence by collagens and the antibody inhibition data implicate the collagen-binding domain of CD206 in the UNO interaction.

Importantly, UNO does not only act as a cellular membrane-docking ligand but is also robustly internalized in CD206-expressing macrophages. This observation agrees with the known physiological role of CD206 as an endocytic receptor for cellular uptake of its ligands, including collagens^34^. Following internalization, CD206 dissociates from its ligands and is recycled back to the cell surface^35^. The ability of UNO peptide to carry the coupled FAM reporter into the MEMs suggests that the peptide can be used for intracellular delivery of therapeutically relevant payloads. In earlier work, targeting the pro-apoptotic peptide _D_[KLAKLAK]_2_ to M2 macrophages was shown to result in improved survival in a mouse syngeneic colon cancer model^22^. Moreover, interferon-α delivery by TIE-2 expressing TAMs in an orthotopic glioma model activated innate and adaptive antitumor responses, which translated into inhibition of cancer progression and near-complete abrogation of metastasis^38^. Tasquinimod, a small molecule antagonist of the S100 calcium-binding protein A9 (S100A9), was recently shown to inhibit MEMs and enhance immunotherapy in prostate and B16 melanoma models^39^. Finally, siRNA knockdown of the endoribonuclease DICER to reprogram M2 macrophages shows therapeutic promise^9,40,41^. MEM-directed delivery with UNO peptide could potentially enhance the efficacy of such approaches.

Our data show that in addition to enabling delivery of molecular payloads, UNO is capable of guiding drug-loaded nanoparticles to MEMs. Cells of monocytic/macrophage lineage have an inherent ability to effectively take up foreign particles, including nanoparticles. However, our polymersome data show that MEMs did not take up these nanoparticles unless they were coated with FAM-UNO. We have previously achieved peptide-mediated delivery of polymersome-encapsulated drugs^42^ and silicon nanoparticle-encapsulated siRNA^43^ to various disease targets.

The potential applications of our MEM-targeting peptide extend beyond therapy. UNO-based imaging agents could be developed into companion diagnostic tests to stratify patients for therapeutic targeting of MEMs and to assess the efficacy of cancer treatments^44^. Moreover, it has been reported that the presence of MEMs in lymph nodes is elevated in early cancer in humans^10^. Here, we showed that UNO homes to MEMs in the lymph nodes, making it potentially suitable for sentinel lymph node imaging.

## Materials and Methods

### Materials

Fluorescent peptides were synthesized by using 5(6)-carboxyfluorescein (FAM) with 6-aminohexanoic acid spacer attached to the N-terminus of the peptide. FAM-Cys-UNO used for nanoparticle coupling was synthesized at Sanford Burnham Prebys Medical Discovery Institute. Peptides were synthesized using Fmoc/t-Bu chemistry on a microwave assisted automated peptide synthesizer (Liberty, CEM Corporation, NC, USA). Peptides were purified by HPLC using 0.1% TFA in acetonitrile-water mixtures to 90–95% purity and validated by Q-TOF mass spectral analysis. All other peptides were purchased from TAG Copenhagen.

### Cell lines and experimental animals

Cell lines were purchased from ATCC (VA, USA). The cells were cultivated in DMEM (Lonza, Belgium) containing 100 IU/mL of penicillin, streptomycin, and 10% of heat-inactivated fetal bovine serum (GE Healthcare, UK) in 5% CO_2_ atmosphere. For animal experimentation athymic nude mice were purchased from HSD (Holland) and Balb/c mice were purchased from Charles River (Wilmington, MA, USA). Animal experimentation protocols were approved by the Estonian Ministry of Agriculture, Committee of Animal Experimentation (Project #42); we confirm that all methods were performed in accordance with the relevant guidelines and regulations.

### *In vivo* phage display

*In vivo* phage display was performed according to protocols approved by the Estonian Ministry of Agriculture, Committee of Animal Experimentation (permit #48). Eight-week old Balb/c mice (Charles River, Wilmington, MA, USA) were injected in the mammary fat pad with 10^6^ 4T1 cells in PBS. Ten days later, the 4T1 mouse and a Balb/c female mouse of the matched age, were injected with 500 µL of a 7 × 10^10^ pfu/mL of purified CX_7_C long-circulating library that bears a mutation on the p17 protein of tail fiber^45^. After 2 h the mice were anesthetized and peritoneal cells were collected, washed by centrifugation, and lysed by hand-held homogenizer in 2 mL of LB + NP40 1%. The phage was amplified in E. coli strain BLT5403 for high throughput sequencing of peptide encoding segment of the phage genomic DNA using Ion Torrent semiconductor sequencing system.

### Extraction and immunostaining of peritoneal cells

For extraction of peritoneal cells, Balb/c mice (healthy and 2 weeks after inoculation of 10^6^ 4T1 cells orthotopically) were anesthetized and intraperitoneally injected with 5 mL of PBS + 1% BSA, the abdomen was massaged to detach macrophages and the peritoneal fluid was collected. This procedure typically yielded 5–9 × 10^6^ cells. The cells were immediately plated on coverslips, let to attach for 2 h, washed with PBS, fixed with PFA for 10 minutes, permeabilized with Triton-X-100 for 10 minutes, and the immunofluorescence protocol was followed, using rat anti-CD206 (Bio-Rad, CA, USA. Product code MCA2235GA) at a 1/100 dilution and anti-rat Alexa Fluor 647 (Invitrogen, CA, USA. Catalog#: A-20991) at a 1/200 dilution.

### Phage biopanning on Raw 267.4 cells

Mouse macrophage cell line Raw 267.4 (ATCC, VA, USA) was cultured in DMEM with FBS and antibiotics. Cells from a 25 cm^2^ flask (~3 × 10^6^ cells) were lifted using a cell scraper, centrifuged, brought to 4 °C and incubated with 0.5 mL of the same purified library used for the *in vivo* experiments (0.5 mL of 7 × 10^10^ pfu/mL) + 0.5 mL of DMEM, at 4 °C overnight. Cells were then washed four times by re-suspending in PBS and placing them in a new tube every time, and finally suspended in LB + 1% NP40, lysed and subjected to high throughput sequencing.

###  Flow cytometry

Peritoneal cells were extracted from Balb/c mice 2 weeks after inoculation of 10^6^ cells orthotopically). Mice were anesthetized and intraperitoneally injected with 5 mL of PBS + 1% BSA, the abdomen was massaged to detach macrophages and the peritoneal fluid was collected. This procedure typically yielded 5–9 × 10^6^ cells. Cells were concentrated to 1 mL in the same peritoneal fluid. For FAM-peptide incubation, the peptides were incubated for 30 minutes at 4 °C. For antibody incubation the cells were incubated with antibody for 1 h at 4 °C. A647 Rat anti-CCR2 (Biolegend, CA, USA. Catalog number 150603) was incubated for 1 h at 4 °C at a dilution of 1/200. FAM-peptides were incubated at a concentration of 0.5 µg/mL, and Rat anti-CD206 (Bio-Rad, CA, USA. Product code MCA2235GA) was incubated at a concentration of 10 µg/mL. Binding of FAM-peptides was monitored with 488 nm channel and CCR2 labeling was assessed in the 647 nm channel using A647 Anti-CCR2 (Biolegend, CA, USA). After incubation of FAM-Peptides or antibodies, the cells were washed 3 times at 4 °C with PBS-T by centrifugation at 250 g for 6 minutes, followed by flow cytometry analysis (Accuri, BD Biosciences, CA, USA).

### Tumor models

Tumor models were induced according to protocols approved by the Estonian Ministry of Agriculture, Committee of Animal Experimentation (permit #48). For the 4T1 breast model, 8-week old female Balb/c mice were injected in the mammary fat pad with 10^6^ cells dispersed in 50 µL of PBS. For homing studies, mice were used 10 days later, when the tumor had reached ~150mm^3^. For MCF-7 breast model, 8-week old female nude mice were implanted with an estrogen pellet, and 1 week later inoculated with 5 × 10^6^ MCF-7 cells in 100 µL of cold Matrigel (BD Biosciences, CA, USA). Mice were used for homing studies three weeks later (tumor volume ~150 mm^3^). For the metastatic gastric carcinoma, 10^6^ MKN45-P cells in PBS were injected intraperitoneally in 8-week old female nude mice. Mice were used for homing studies two weeks later. For the metastatic melanoma model, 2 × 10^5^ B16F10 cells were injected intravenously in 100 µL of PBS in C57 BL6 mice. The mice were used for homing studies 10 days after.

For the angiogenic glioblastoma model, WT-GBM^24^, 7 × 10^5^ cells were stereotactically injected in the right striatum of Fox/Nu mice 2 mm lateral and 2 mm posterior of bregma at depth of 2.5 mm. The mice were used for homing studies 6-7 days after the injection.

### Homing studies and Immunofluorescence

For free peptide homing studies, mice were injected intravenously with 30nmoles of FAM-peptides in 100 µL of PBS. For FAM-UNO-NP-PTX homing studies, the MCF-7 breast tumor mice were injected intravenously with 1 mg of polymer in 100 µL pf PBS and the particles were allowed to circulate for 6 h. For tissue collection, the mice were anesthetized and the tissues were collected and immediately placed in cold 4% PFA and left overnight at 4 °C. Tissues were washed with PBS for 2 h at room temperature and cryoprotected with sucrose 15% at 4 °C overnight and then in sucrose 30% at 4 °C overnight. The tumor and the control organs were placed together in the same block with OCT compound (Leica Biosystems, Wetzlar, Germany), snap-frozen in isopentane, and sectioned at 10μm. FAM was detected using Rabbit anti-FITC (Invitrogen, CA, USA. Catalog # A-889) at a dilution of 1/100 and using Alexa Fluor-546 anti-rabbit antibody (Invitrogen, Ca, USA. Catalog # A-11035) at a dilution of 1/200. CD31 was detected using rat anti-mouse CD31 (BD Biosciences, CA, USA. Catalog #: 553370), TIE2 was detected using rat anti-mouse TIE2 (Biolegend, CA, USA. Catalog # 124001), CD209 was detected using rat anti-mouse CD209 (Biolegend, CA, USA. Catalog # 147802), HLA ABC was detected using rat anti Human HLA ABC (Bio-Rad, CA, USA. Product code MCA485G), and CD206 was detected using rat anti-mouse CD206 (Bio-Rad, CA, USA. Product code MCA2235GA); using 1/100 primary antibody dilution and Alexa Fluor-647 anti-Rat antibody (Invitrogen, CA, USA. Catalog# 21247) at 1/200 dilution. To quantify FAM signal from confocal images, the green channel of images taken by using 20× magnification objective was opened with ImageJ, converted to 8-bit grayscale and the integrated pixel intensity was measured. Different regions of tumors from three different mice were used. These measures were normalized to the number of cells in each image; to count the number of DAPI^+^ cells, the blue channel was opened with ImageJ, converted to 8 bit grayscale, thresholded and cells were counted using the analyze particle tool. The ratio: FAM signal/number of DAPI^+^ cells was graphed and is shown in Fig. 2i. The same procedure was used to count the number of CD206^+^ cells.

### Nanoparticle synthesis, loading and characterization

Polyethylene glycol-polycaprolactone (PEG(5000)-PCL(10000)) and maleimide-polyethylene glycol-polycaprolactone (Mal-PEG(5000)-PCL(10000)) were purchased from Advanced Polymer Materials Inc. (Canada). The Mal-PEG(5000)-PCL(10000) copolymer (10 mg, 0.7 µmol) was dissolved in 1 mL of nitrogen-purged dimethylformamide. Two equivalents of FAM-Cys-UNO or FAM-Cys, wherein the cysteine is used for conjugation, were dissolved in 0.5 mL of nitrogen-purged dimethylformamide, and 2 µL of triethylamine were added to the solution. The mixture was mixed overnight at room temperature. The solution was dialyzed against water using a cellulose membrane of 3 KDa MWCO (Thermo Scientific, USA). The resulting suspension was freeze-dried and a yellow powder was obtained. To generate Paclitaxel (PTX) loaded, FAM-UNO or FAM labeled polymersomes, denoted as FAM-UNO-NP-PTX and FAM-NP-PTX respectively: 1 mg of FAM-PEG-PCL or FAM-UNO-PEG-PCL copolymer was mixed with 9 mg of unlabeled PEG-PCL copolymer dissolved in 2 mL of acetone in a glass vial, and 0.75 µmol of PTX dissolved in MeOH were added to the copolymer solution. The solution was then dried under vacuum to allow for the formation of the polymer-drug film. The films were hydrated with 1 mL of PBS (pH 7.4) and sonicated for 2 h. The amount of FAM-labeled peptides in polymersomes was quantified by fluorometry (FlexStation II, Molecular Devices) at 485/520 nm. Dynamic Light Scattering (“DLS”; Zetasizer Nano ZS, Malvern Instruments, USA) was used to assess the polydispersity and average size of the polymersomes. Transmission electron microscopy (TEM) was used to assess the size, surface topology and morphology of assembled vesicles. Briefly, polymersomes dispersed in PBS at pH 7.4 were deposited onto copper grids at 1 mg/mL, stained with 0.75% phosphotungstic acid (pH 7), air-dried, and imaged by TEM (Tecnai 10, Philips, Netherlands).

To evaluate stability of FAM-UNO on FAM-UNO-NP-PTX in serum: 200 µL of blood were extracted from the tail vein of a mouse bearing orthotopic 4T1 breast tumor (10 days after inoculation of 10^6^ cells) in a blood collection tube (BD vacutainer, REF: 368494), plasma was separated by centrifugation (300 g for 7 minutes). Later, 150 µL of FAM-UNO-NP-PTX in PBS were mixed with 150 µL of serum and incubated for 6 h at 37 °C with shaking, then particles were washed by 3 centrifugation cycles (21000 g for 30 minutes), and redispersed in 150 µL of PBS to obtain “FAM-UNO-NP-PTX-Serum”. Then, 150 µL of the original FAM-UNO-NP-PTX in PBS and 150 µL of FAM-UNO-NP-PTX-Serum were placed in a 96 well plate and the fluorescence was measured using a FlexStation 3 Multi-Mode Microplate Reader (Molecular Devices) using 490 nm excitation and collecting at 520 nm.

### Fluorescence anisotropy

The stocks of fluorescent ligands (FAM-UNO, FAM-CSPGAK and FAM-CPMTDNE) in mQ water were stored at −20 °C and diluted with incubation buffer IB (10 mMNa-HEPES, 150 mM NaCl, 1 mM CaCl_2_, 0.1% Pluronic F-127, pH 7.4) on the day of experiment. The concentration of fluorescent ligands was determined by absorbance of FAM (ε_495_ = 75000 M^−1^ cm^−1^). The recombinant mouse macrophage mannose receptor rmMMR/CD206 (R&D Systems, catalog number: 2535-MM-050) and recombinant mouse CD163 (R&D Systems, catalog number: 7435-CD-050) were reconstituted at 1 mg/ml in IB, aliquoted and stored at −20 °C. To generate linearized version of cyclic FAM-UNO peptide, the peptide at 1 mM concentration was preincubated for 24 h at room temperature in a dark place with 3 mM DTT and diluted with IB 2 × 10^5^ times before the FA assay. The control experiment with FAM-CPMTDNE peptide (Fig. S16) was performed to rule out the possibility of disulphide bond formation, which could affect the binding properties of linear peptides. Initially, the peptide was preincubated in DMSO for 4 days at room temperature to enhance the disulphide bond formation, and then diluted in IB buffer with 3 mM DTT, and the disappearance of preformed dimer was observed as a change in FA signal. In all experiments, FA signal measurements were done under pseudo first-order conditions with 50 nM singly labelled FAM peptides and 1 µM proteins. Black 384-well round bottom polystyrene NBS surface microplates (Corning, Product No. 3676) that were found to give optimal results for our assays (low background of fluorescence and low adsorption of ligands onto plastic surface) were used in all FA experiments.

Assays were carried out in kinetic mode in a total volume of 30 μl at 25 °C on the PHERAstar (BMG Labtech, Germany) microplate reader using an optical module with excitation and emission filters of 485 nm (slit 10 nm) and 520 nm (slit 10 nm), respectively. Dual emission detection mode allows simultaneous recording of intensities that are parallel (*I*_||_) and perpendicular (*I*_⊥_) to the plane of excitation light. Sensitivities of channels (G factor) were corrected with gain adjustment of the photomultiplier tubes (PMTs) using fluorescein as a standard. The background fluorescence (receptor and buffer components in the absence of fluorescent ligand) was subtracted independently from all intensity channels. FA signals at time *t* after initiation of binding reaction was calculated as parameters *r*(*t*) from the equation:

$$r(t)=\frac{I{(t)}_{{\rm{II}}}-I{(t)}_{\perp }}{I{(t)}_{{\rm{II}}}+2\cdot I{(t)}_{\perp }}$$

### Liquid-chromatography mass spectrometry (LC-MS) analysis

High-performance liquid chromatography (Agilent 1200 series, USA) – mass spectrometry (Sciex Q-Trap 4500, Canada) analysis was done using a C18 column (Kinetex 2.6 µm EVO C18 100 × 4.6 mm, Phenomenex, USA). Ionization was performed at 300 °C with declustering potential set 30 V. Chromatography gradient started with 2 min 5% acetonitrile in water, followed by linear increase to 95% acetonitrile in 10 min and finally 10 min isocratic flow of 95% acetonitrile in water. Both eluents, water and acetonitrile, contained 0.2% formic acid. Flow rate was 0.3 ml/min. Column temperature was maintained at 40 °C. Autosampler temperature was set to 37 °C.

Retention times and m/z signals of FAM-UNO, were determined from 30 µM FAM-UNO solution in PBS. Tumor lysate was split into two aliquotes of 200 µL in each. From one aliquot, 20 µL were injected and the LC-MS profile obtained. The second aliquot was mixed with 50 µL of 30 µM FAM-UNO in PBS and injected within 1 min to LC-MS and the LC-MS profile obtained.

### Statistical analysis

All data represents mean value + SEM. Significance analysis were done using Statistica 8.0 software, using one-way ANOVA.

## Electronic supplementary material

Supplementary Information

Supplementary Video 1

## References

[1] Lewis CE, Harney AS, Pollard JW (2016). The Multifaceted Role of Perivascular Macrophages in Tumors. Cancer Cell.

[2] Williams CB, Yeh ES, Soloff AC (2016). Tumor-associated macrophages: unwitting accomplices in breast cancer malignancy. npj Breast Cancer.

[3] Zhou W (2015). Periostin secreted by glioblastoma stem cells recruits M2 tumour-associated macrophages and promotes malignant growth. Nat. Cell Biol..

[4] Hambardzumyan D, Gutmann DH, Kettenmann H (2015). The role of microglia and macrophages in glioma maintenance and progression. Nat. Neurosci..

[5] Noy R, Pollard JW (2014). Tumor-Associated Macrophages: From Mechanisms to Therapy. Immunity.

[6] Hughes R (2015). Perivascular M2 macrophages stimulate tumor relapse after chemotherapy. Cancer Res..

[7] Pollard JW (2004). Tumour-educated macrophages promote tumour progression and metastasis. Nat. Rev. Cancer.

[8] De Palma M, Lewis CE (2011). Cancer: Macrophages limit chemotherapy. Nature.

[9] Baer C (2016). Suppression of microRNA activity amplifies IFN-γ-induced macrophage activation and promotes anti-tumour immunity. Nat. Cell Biol..

[10] Azad AK (2015). -Tilmanocept, a New Radiopharmaceutical Tracer for Cancer Sentinel Lymph Nodes, Binds to the Mannose Receptor (CD206). J. Immunol..

[11] Mazzieri R (2011). Targeting the ANG2/TIE2 Axis Inhibits Tumor Growth and Metastasis by Impairing Angiogenesis and Disabling Rebounds of Proangiogenic Myeloid Cells. Cancer Cell.

[12] Seo JW (2014). 64 Cu-Labeled LyP-1-Dendrimer for PET-CT Imaging of Atherosclerotic Plaque. Bioconjug. Chem..

[13] Sharma, S. *et al*. Tumor-Penetrating Nanosystem Strongly Suppresses Breast TumorGrowth. *Nano Lett*. acs.nanolett.6b03815 10.1021/acs.nanolett.6b03815 (2017). 10.1021/acs.nanolett.6b03815 PMC5819594 28178415

[14] Paasonen L (2016). New p32/gC1qR Ligands for Targeted Tumor Drug Delivery. ChemBioChem.

[15] Sharma, G. *et al*. Depletion of Tumor-Associated Macrophages with Clodronate-Loaded Plga Nanoparticles. *Nano Life* (2013).

[16] Laakkonen P (2004). Antitumor activity of a homing peptide that targets tumor lymphatics and tumor cells. Proc. Natl. Acad. Sci. USA.

[17] Geijtenbeek TBH (2000). Identification of DC-SIGN, a novel dendritic cell-specific ICAM-3 receptor that supports primary immune responses. Cell.

[18] Jameson B (2002). Expression of DC-SIGN by dendritic cells of intestinal and genital mucosae in humans and rhesus macaques. J. Virol..

[19] Movahedi K (2012). Nanobody-based targeting of the macrophage mannose receptor for effective *in vivo* imaging of tumor-associated macrophages. Cancer Res..

[20] Blykers A (2015). PET Imaging of Macrophage Mannose Receptor-Expressing Macrophages in Tumor Stroma Using 18F-Radiolabeled Camelid Single-Domain Antibody Fragments. J. Nucl. Med..

[21] Jaynes, J. M. *et al*. Peptides Having Anti-Inflammatory Properties. *US Patent*, *20160101150* (2016).

[22] Cieslewicz M (2013). Targeted delivery of proapoptotic peptides to tumor-associated macrophages improves survival. Proc. Natl. Acad. Sci. USA.

[23] Laakkonen P, Porkka K, Hoffman JA, Ruoslahti E (2002). A tumor-homing peptide with a targeting specificity related to lymphatic vessels. Nat. Med..

[24] Blouw B (2003). The hypoxic response of tumors is dependent on their microenvironment. Cancer Cell.

[25] Yonemura Y (2001). Inhibition of peritoneal dissemination in human gastric cancer by MMP-7-specific antisense oligonucleotide. J Exp Clin Cancer Res.

[26] Napper CE, Drickamer K, Taylor ME (2006). Collagen binding by the mannose receptor mediated through the fibronectin type II domain. Biochem. J..

[27] Fernandes AP, Holmgren A (2004). Glutaredoxins: glutathione-dependent redox enzymes with functions far beyond a simple thioredoxin backup system. Antioxid. Redox Signal..

[28] Nagy P (2013). Kinetics and mechanisms of thiol-disulfide exchange covering direct substitution and thiol oxidation-mediated pathways. Antioxid. Redox Signal..

[29] Khramtsov VV, Gillies R (2014). J. Janus-Faced Tumor Microenvironment and Redox. Antioxid. Redox Signal..

[30] Gautier EL (2012). Gene-expression profiles and transcriptional regulatory pathways that underlie the identity and diversity of mouse tissue macrophages. Nat. Immunol..

[31] Xin H (2012). Anti-glioblastoma efficacy and safety of paclitaxel-loading Angiopep-conjugated dual targeting PEG-PCL nanoparticles. Biomaterials.

[32] Fogal, V., Zhang, L., Krajewski, S. & Ruoslahti, E. Mitochondrial/Cell-Surface Protein p32/gC1qR as a Molecular Target in Tumor Cells and Tumor Stroma. *Cancer Res*. **68** (2008). 10.1158/0008-5472.CAN-07-6752 PMC2562323 18757437

[33] Martinez-Pomares L, Linehan Sa, Taylor PR, Gordon S (2001). Binding properties of the mannose receptor. Immunobiology.

[34] Martinez-Pomares L (2012). The mannose receptor. J. Leukoc. Biol..

[35] Magnusson S, Berg T (1989). Extremely rapid endocytosis mediated by the mannose receptor of sinusoidal endothelial rat liver cells. Biochem. J..

[36] Madsen DH (2013). M2-like macrophages are responsible for collagen degradation through a mannose receptor-mediated pathway. J. Cell Biol..

[37] Martinez-Pomares L (2006). Carbohydrate-independent recognition of collagens by the macrophage mannose receptor. Eur. J. Immunol..

[38] De Palma M (2008). Tumor-Targeted Interferon-?? Delivery by Tie2-Expressing Monocytes Inhibits Tumor Growth and Metastasis. Cancer Cell.

[39] Shen L (2015). Tasquinimod modulates suppressive myeloid cells and enhances cancer immunotherapies in murine models. Cancer Immunol. Res..

[40] Squadrito ML, De Palma M (2016). DICERing macrophages for reprogramming TAMs. Cell Cycle.

[41] Martello G (2010). A microRNA targeting dicer for metastasis control. Cell.

[42] Simon-Gracia L (2016). iRGD peptide conjugation potentiates intraperitoneal tumor delivery of paclitaxel with polymersomes. Biomaterials.

[43] Mann AP (2016). A peptide for targeted, systemic delivery of imaging and therapeutic compounds into acute brain injuries. Nat. Commun..

[44] Lammers T, Rizzo LY, Storm G, Kiessling F (2012). Personalized nanomedicine. Clinical Cancer Research.

[45] Ludtke JJ, Sololoff AV, Wong SC, Zhang G (2007). & Wolff, J. a. *In vivo* selection and validation of liver-specific ligands using a new T7 phage peptide display system. Drug Deliv..

[46] Uchida M (2011). Protein cage nanoparticles bearing the LyP-1 peptide for enhanced imaging of macrophage-rich vascular lesions. ACS Nano.

[47] Zhang F (2012). Imaging tumor-induced sentinel lymph node lymphangiogenesis with LyP-1 peptide. Amino Acids.

