# Supplementary material for: Precision Targeting of Tumor Macrophages with a CD206 Binding Peptide
Source: Sci Rep. 2017 Nov 7;7:14655. doi: 10.1038/s41598-017-14709-x (PMC5676682; doi:10.1038/s41598-017-14709-x)
Supplement: Supplementary file 1 — Supplementary Information [file 41598_2017_14709_MOESM1_ESM.doc]

**PRECISION TARGETING OF TUMOR MACROPHAGES WITH A CD206 BINDING PEPTIDE**

Pablo Scodeller* ^a,b^, Lorena Simon-Gracia ^a^, Sergei Kopanchuk^c^, Allan Tobi ^a^, Kalle Kilk^d^, Pille Säälik ^a^, Kaarel Kurm ^a^, Mario Leonardo Squadrito^e^, Venkata Ramana Kotamraju^b^, Ago Rinken^c^, Michele De Palma^e^, Erkki Ruoslahti ^b,f^, Tambet Teesalu ^a,b,f^*

^a^ Laboratory of Cancer Biology, Institute of Biomedicine and Translational Medicine, University of Tartu, Ravila 14B, Tartu, 50411, Estonia

^b^ Cancer Research Center, Sanford Burnham Prebys Medical Discovery Institute, 10901 N. Torrey Pines Road, La Jolla, 92097 California, USA

^c^ Institute of Chemistry, University of Tartu, Ravila 14, Tartu, 50411, Estonia

^d^Department of Biochemistry, Institute of Biomedicine and Translational Medicine, University of Tartu, Ravila 14B, Tartu, 50411, Estonia

^e^School of Life Sciences, Ecole Polytechnique Fédérale de Lausanne (EPFL), (Swiss Federal Institute of Technology, Lausanne), CH-1015 Lausanne, Switzerland

^f^ Center for Nanomedicine and Department of Cell, Molecular and Developmental Biology, University of California, Santa Barbara Santa Barbara, 93106 California, USA

*Corresponding authors:

Tambet Teesalu: Laboratory of Cancer Biology, Institute of Biomedicine, Centre of Excellence for Translational Medicine, University of Tartu, Ravila 14b, 50411 Tartu, Estonia. E-mail address: tambet.teesalu@ut.ee

Pablo Scodeller: Laboratory of Cancer Biology, Institute of Biomedicine, Centre of Excellence for Translational Medicine, University of Tartu, Ravila 14b, 50411 Tartu, Estonia. E-mail address: pablo.david.scodeller@ut.ee


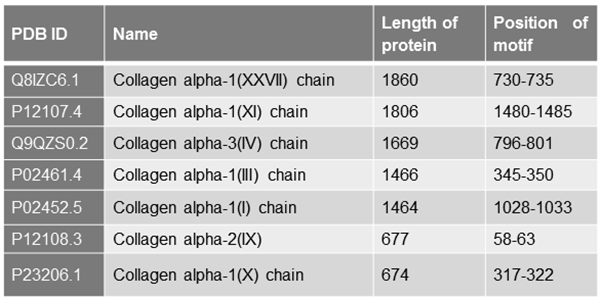


**Table 1.** Collagens containing a GSPGAK motif.


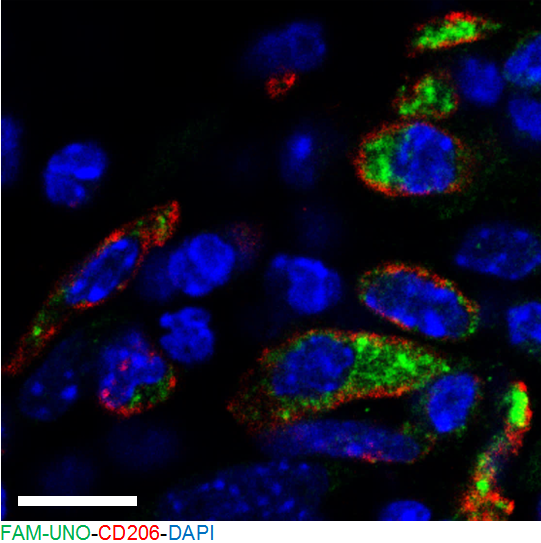


**Fig. S1. Confocal microscopy using low optical thickness and high magnification, of FAM-UNO in MEMs**. Thirty nmoles of FAM-UNO were injected intravenously into 4T1 tumor-bearing mice and allowed to circulate for 2 hours. Mice were then sacrificed and tumors were analyzed by immunofluorescence using rabbit anti-FAM (green) and rat anti-CD206. Sections were imaged using 0.9µm optical thickness at 63X. Scale bar: 10µm. Representative image from n=3 mice.

**VIDEO1. 3D reconstruction of FAM-UNO in MEM**

Thirty nmoles of FAM-UNO were injected intravenously into 4T1 tumor-bearing mice and allowed to circulate for 2 hours. Mice were then sacrificed and tumors were analyzed by immunofluorescence using rabbit anti-FAM (green) and rat anti-CD206 (red). Then, Z-stack images were taken using 0.9µm optical thickness at 63X and spaced 1µm apart and three dimensionally reconstructed using ImageJ.

**
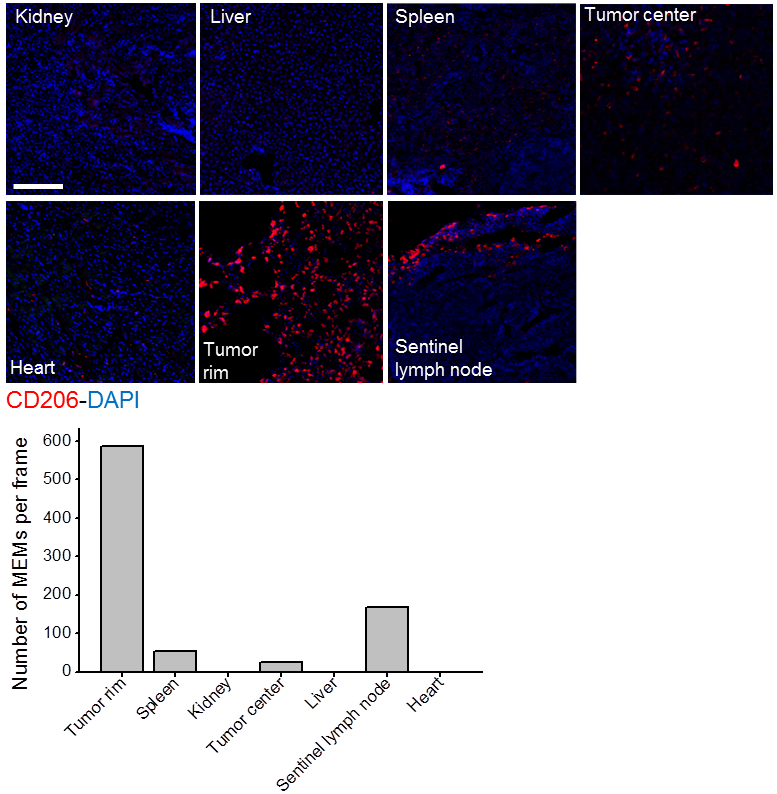
**

**Fig. S2. MEMs highly overpopulate the tumor rim and are abundant in a sentinel lymph node.** Tumor and organs from a 4T1 tumor mouse were stained for CD206, using rat anti-CD206 (red) and counterstained with DAPI (blue). Scale bar: 100µm. Representative images from n=3 mice.


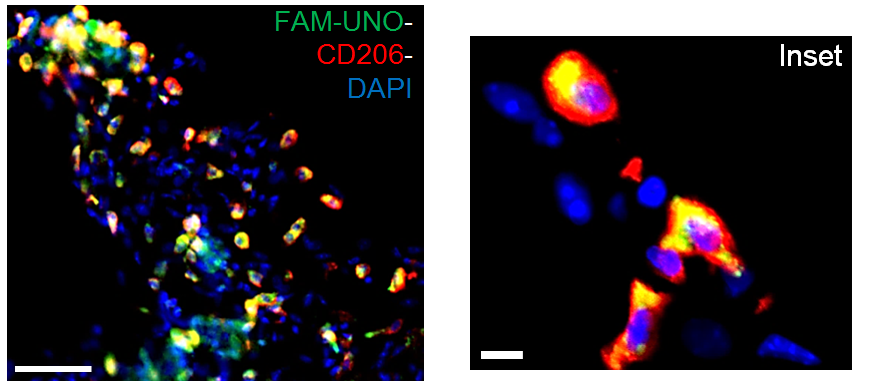


**Fig. S3.** **FAM-UNO accumulates in MEMs after 12-hour circulation in 4T1 breast cancer.** Thirty nmoles of FAM-UNO were injected intravenously in mice bearing 4T1 tumors, 10 days after orthotopic inoculation of 10^6^ cells. Peptide was allowed to circulate for 12 hours . Mice were then sacrificed, and tumor and tissues analyzed by immunofluorescence using rabbit anti-FAM (green) together with rat anti-CD206 (red) and counterstained with DAPI (blue). Scale bars: 50µm (left) and 10µm (right). Representative images from n=3 mice.


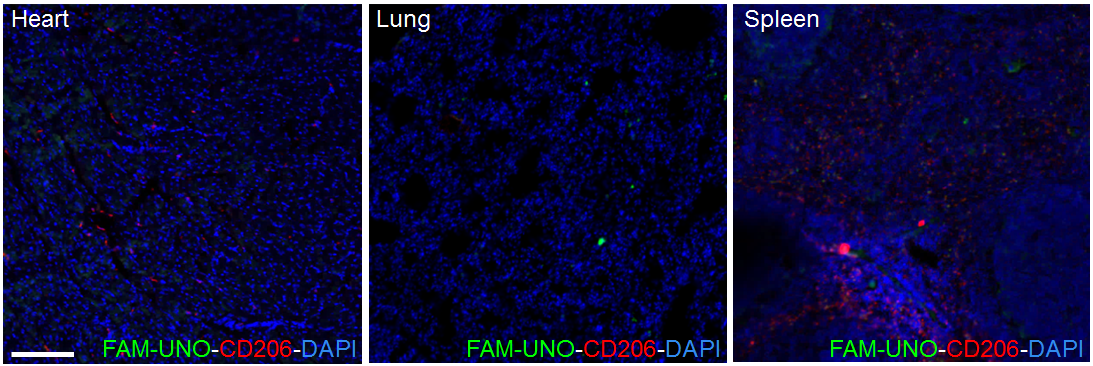


**Fig. S4.** **FAM-UNO does not accumulate in heart, lung and spleen.** Heart, lung and spleen of Fig. 2. Scale bar: 100µm. Representative images from n=3 mice.


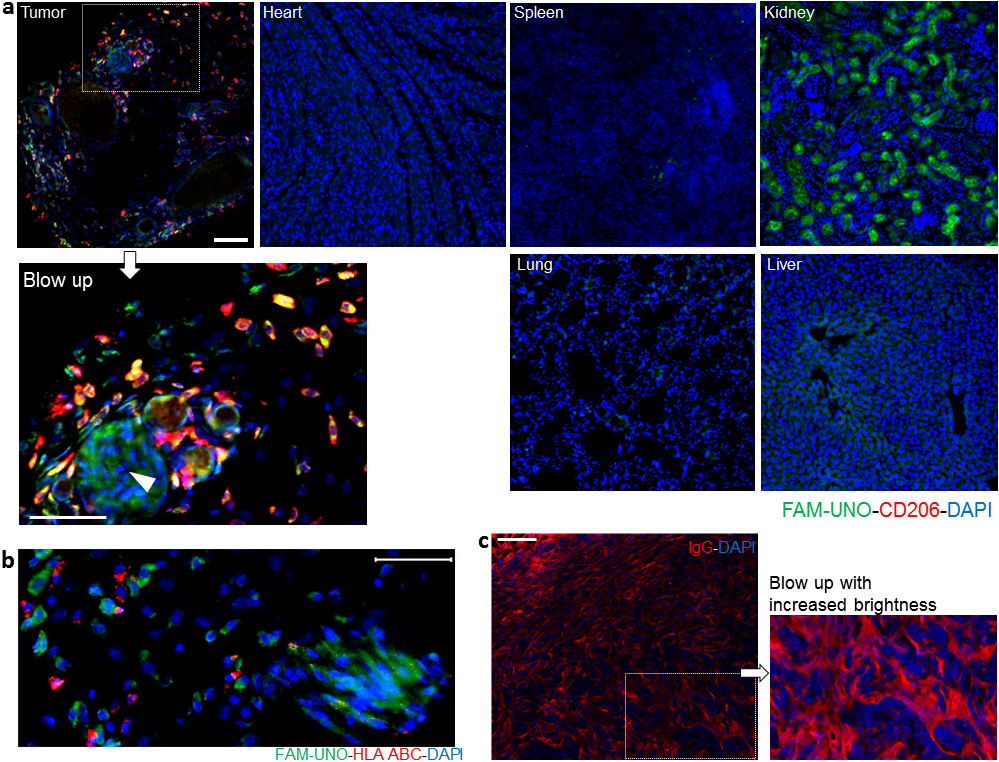


**Fig. S5. FAM-UNO accumulates in MEMs in MCF-7 breast cancer.** **A,** Thirty nmoles of FAM-UNO were injected intravenously in mice bearing MCF-7 tumors, 21 days after orthotopic inoculation of 5x10^6^ cells. The peptide was allowed to circulate for 2 hours, the mouse was then sacrificed, and the tumor and tissues were analyzed by immunofluorescence using rabbit anti-FAM (green), rat anti-CD206 (red) and counterstained with DAPI (blue). All images were taken with the same imaging conditions. Scale bar: 100µm and 50µm (blow up). **B,** FAM-UNO does not home to cancer cells**.** The tumor tissue from panel A was analyzed by immunofluorescence using rabbit anti-FAM (green), rat anti-HLA ABC (red) and counterstained with DAPI (blue). Scale bar: 50µm. **C,** MCF-7 tumors are leaky. Tumor sections were immunostained for endogenous mouse IgG and counterstained with DAPI using the same staining and imaging conditions as in Fig. S11. Scale bar: 100µm. Representative images from n=3 mice.


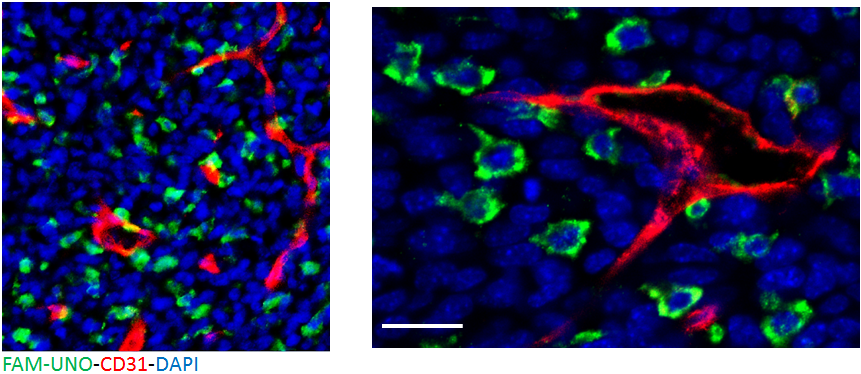


**Fig. S6. FAM-UNO is internalized by perivascular cells in WT-GBM tumor.** Thirty nmoles of FAM-UNO were injected intravenously in tumor mice. Peptide was allowed to circulate for 2 hours. Mice were then sacrificed and tumor and tissues were analyzed by immunofluorescence using rabbit anti-FAM (green) together with rat anti-CD31 (blood vessels; red) and counterstained with DAPI. Scale bar: 20µm. Representative images from n=3 mice.


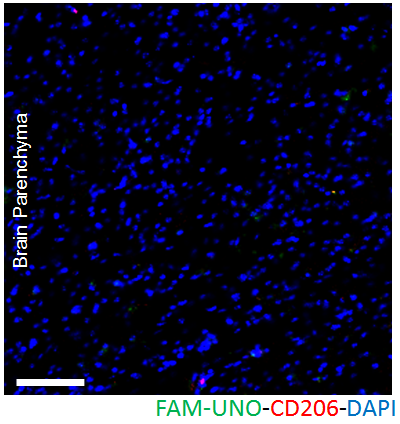


**Fig. S7. FAM-UNO does not home to brain parenchyma.** The contralateral side of the brain shown in Fig. 3A stained and imaged under the same conditions as in Fig. 3A. Scale bar: 50µm. Representative images from n=3 mice.


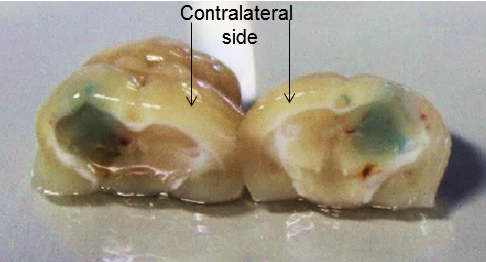


**Fig. S8. WT-GBM tumors are leaky.** Seven days after orthotopic implantation of WT-GBM cells, mice were intravenously injected with 100µL of 0.5% Evans blue solution in PBS and allowed to circulate for 1 h. Mice were then perfused with PBS and brains were extracted and photographed. Representative images from n=3 mice.


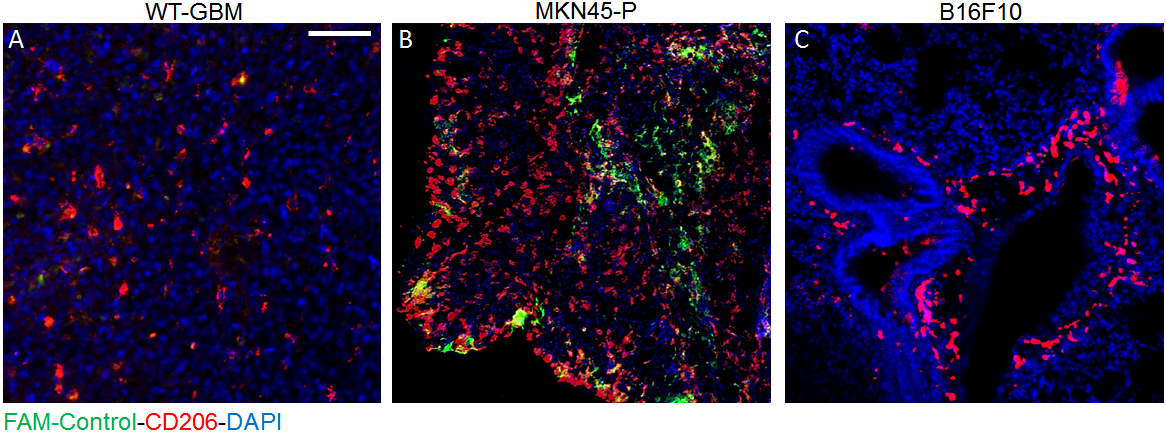


**Fig. S9. FAM-control peptide (CRKQGEAKC) does not accumulate in MEMs in gastric carcinoma, glioblastoma and melanoma tumors.** Thirty nmoles of FAM-CRKQGEAKC were injected intravenously in mice. The peptide was allowed to circulate for 2 hours. Mice were then sacrificed, and tumor and tissues analyzed by immunofluorescence using rabbit anti-FAM (green) together with rat anti-CD206 (red), and counterstained with DAPI. FAM-CRKQGEAKC injections were made at the same day after tumor inoculation as FAM-UNO injections in Figure 4, and the images were taken under the same conditions. Scale bar: 100µm. Representative images from n=3 mice.


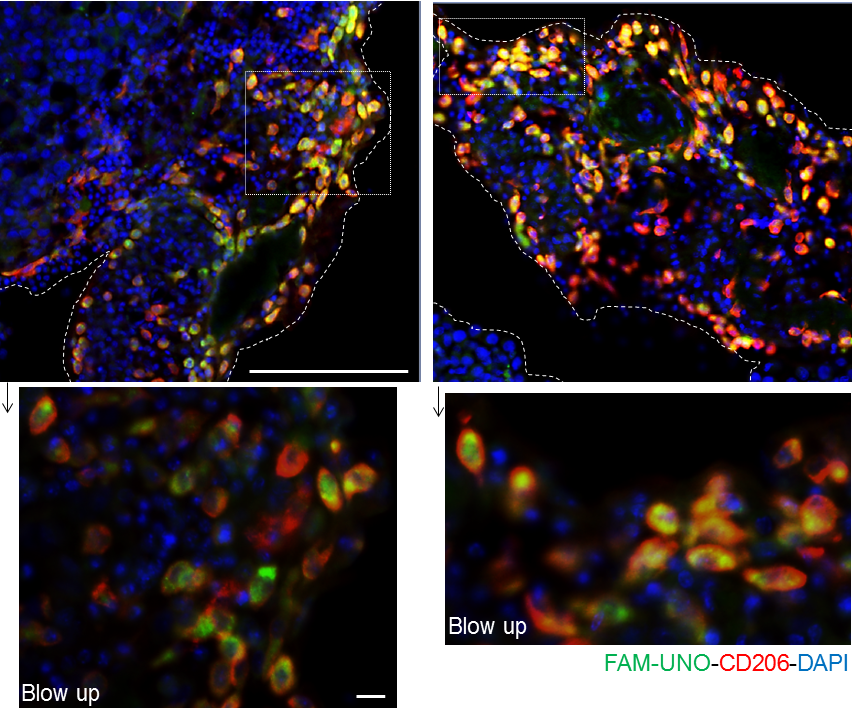


**Fig. S10. FAM-UNO homes to MEMs in the rim of MKN45-P tumor nodules.** Thirty nmoles of FAM-UNO were injected intravenously in mice. The peptide was allowed to circulate for 2 hours. The mice were then sacrificed and the tumors and tissues were analyzed by immunofluorescence using rabbit anti-FAM (green) together with rat anti-CD206 (red), and counterstained with DAPI. Scale bar: 100µm and 10µm (blow up). Representative images from n=3 mice.


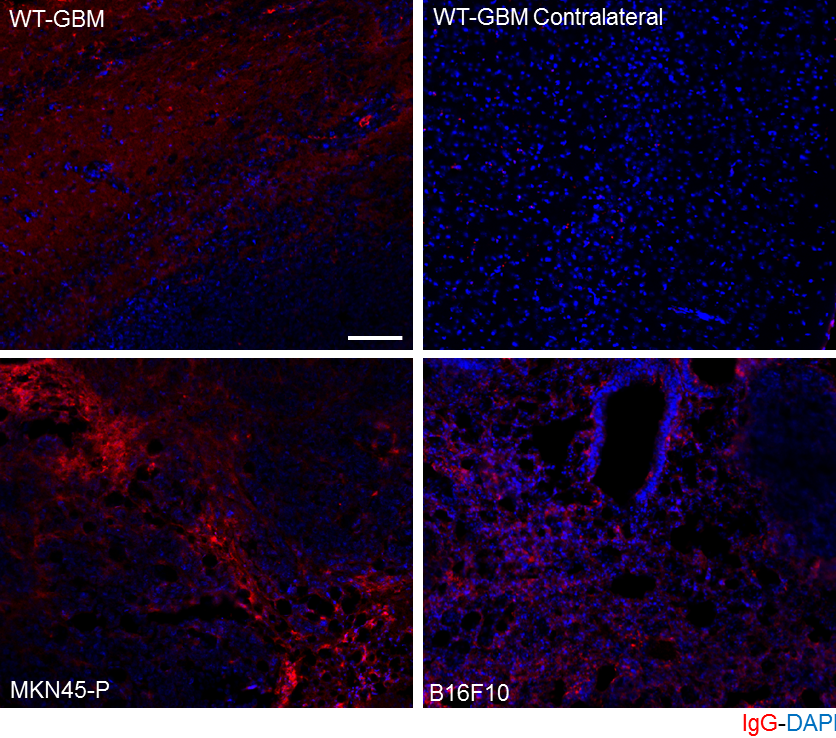


**Fig. S11. Endogenous IgG immunostaining (red) of WT-GBM, MKN45-P and B16F10 tumors. Scale bar: 100µm.** Representative images from n=3 mice.


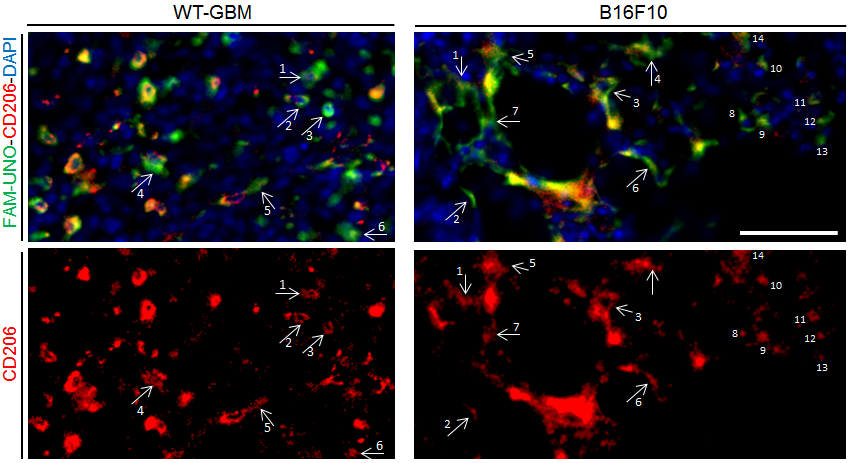


**Fig. S12.** Coincidence between FAM-UNO^+^ and CD206^+^ structures in WT-GBM and B16F10 tumors, where CD206 (red) channel is shown separately. Arrows point to regions that at first sight seem green in the merged image. Scale bar: 50µm. Representative images from n=3 mice.


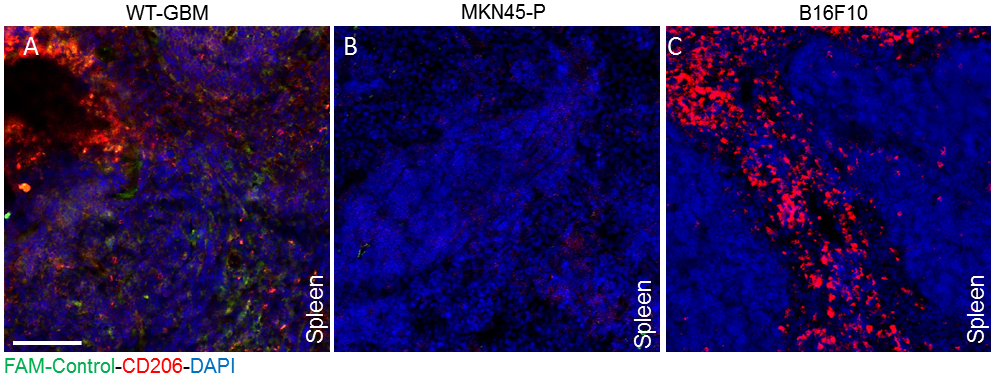


**Fig. S13.** **FAM-UNO does not accumulate in the spleen of gastric carcinoma, glioblastoma or melanoma tumor mice.** Thirty nmoles of FAM-UNO were injected intravenously in mice. The peptide was allowed to circulate for 2 hours. The mice were then sacrificed, and the tumors and tissues were analyzed by immunofluorescence using rabbit anti-FAM (green) together with rat anti-CD206 (red), and counterstained with DAPI. All images were taken under the same conditions as those of Fig. 4. Scale bar: 100µm. Representative images from n=3 mice.


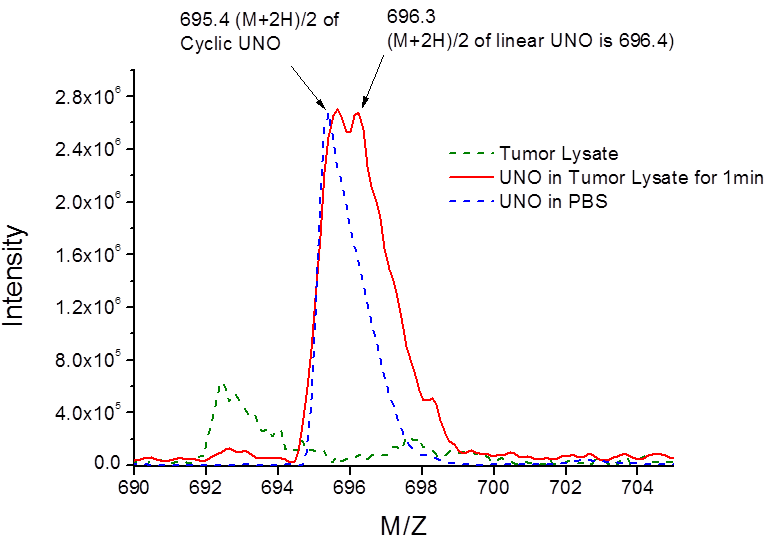


**Fig S14**. **FAM-UNO gets linearized in presence of orthotopic 4T1 tumor lysate.**

Mass spectra region m/z 690-705 for retention period 11.5-13.0 min, which corresponds to the retention of FAM-UNO (blue dotted line). [M+2H]2+ with m/z=695.4 was chosen as the representative signal for having the highest signal to noise ratio of all FAM-UNO related signals. Orthotopic 4T1 tumor lysate (green dotted line) lacks constituents with m/z 694-700 eluting at the same time frame. On the first minute upon FAM-UNO addition to tumor lysate the signal from the peptide (red solid line) is broadened, due to appearance of another peak with maximum intensity at 696.3. The new signal corresponds to a compound with two additional hydrogens compared with cyclic FAM-UNO.


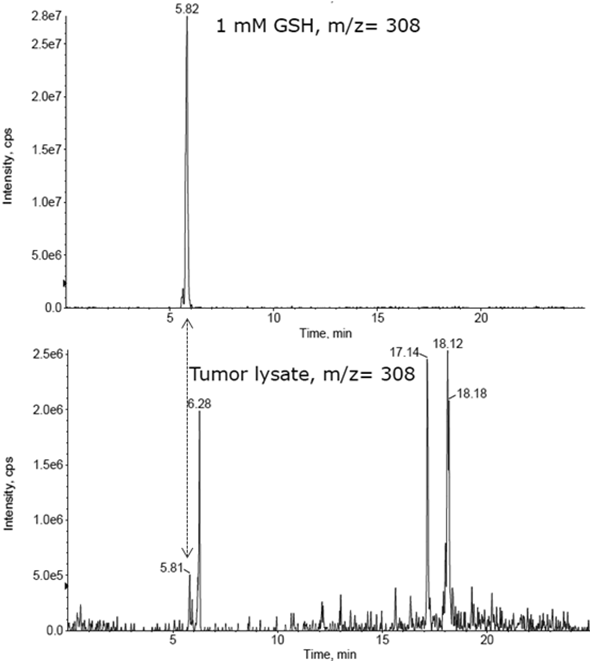


**Fig. S15.** **Presence of Glutathione (GSH) in orthotopic 4T1 tumor lysate.** Upper panel: chromatogram of m/z = 308 (this mass corresponds to GSH molecular ion [M+H]+) on 1 mM GSH solution, showing the retention time (5.8 min) for GSH. Lower panel: chromatogram of m/z = 308 on tumor lysate, showing a retention peak at 5.8 min, which is the same retention time as for pure GSH.


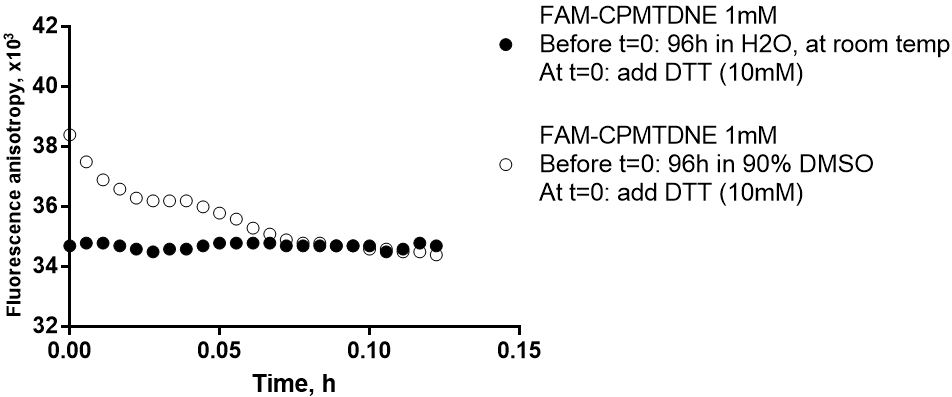


**Fig. S16.** **FAM-CPMTDNE does not dimerize in water**. FAM-CPMTDNE peptide was preincubated in 90% DMSO for 4 days at room temperature to enhance the disulphide bond formation (open circles). The observed higher FA signal in comparison with peptide kept in H_2_O (filled circles) is caused by a rotational mobility decrease due to a mass increase upon dimer formation in DMSO. Addition of DTT caused time-dependent decrease of FA signal, linked to the dissociation of the dimer to FAM-CPMTDNE monomers.


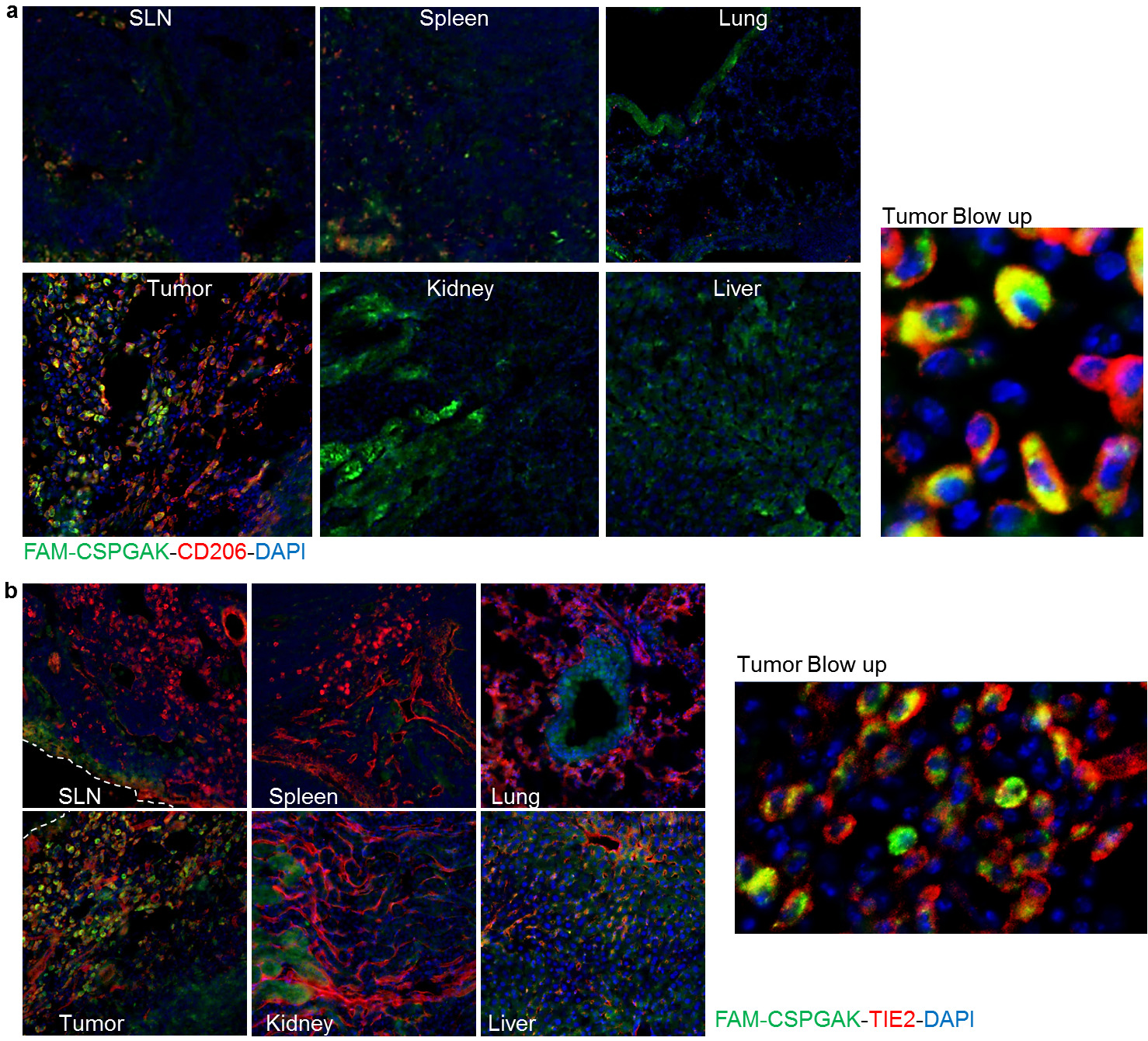


**Fig. S17. FAM-CSPGAK accumulates in MEMs.** Thirty nmoles of FAM-CSPGAK were injected intravenously in mice bearing 4T1 tumors, 10 days after orthotopic inoculation of 10^6^ Cells. Peptide was let to circulate for two hours, mice were then sacked and tumor and tissues analyzed by immunofluorescence using rabbit anti-FAM (green) together with rat anti-CD206(red) and counterstained with DAPI (Blue). All images were taken with the same imaging conditions. Representative images from n=3 mice.


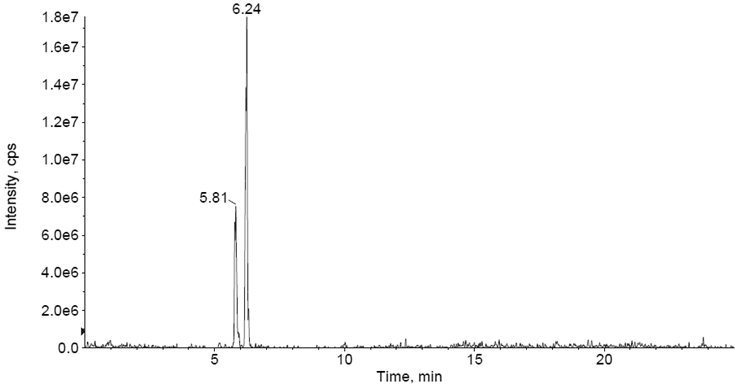


**Fig. S18.** **Presence of Glutathione (GSH) in i.p fluid of orthotopic 4T1 tumor bearing mouse.** Chromatogram of m/z = 308 on i.p fluid from 4T1 tumor-bearing mouse, showing a retention peak at 5.8 min, which is the same retention time as for pure GSH (see upper panel of Fig. S15).


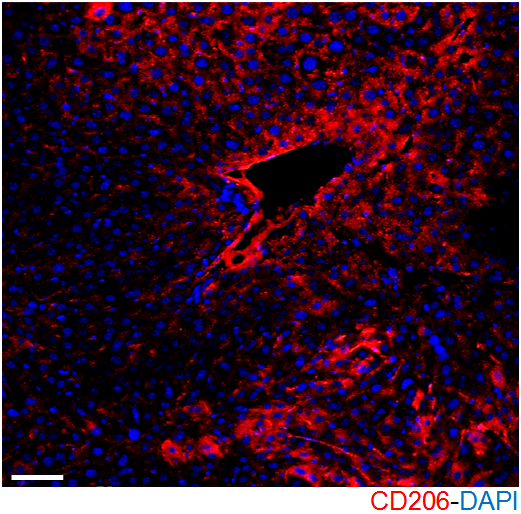


**Fig. S19**. A liver section from a 4T1 tumor mouse imaged using higher gain than in Fig.2. Scale bar: 50µm. Representative images from n=3 mice.


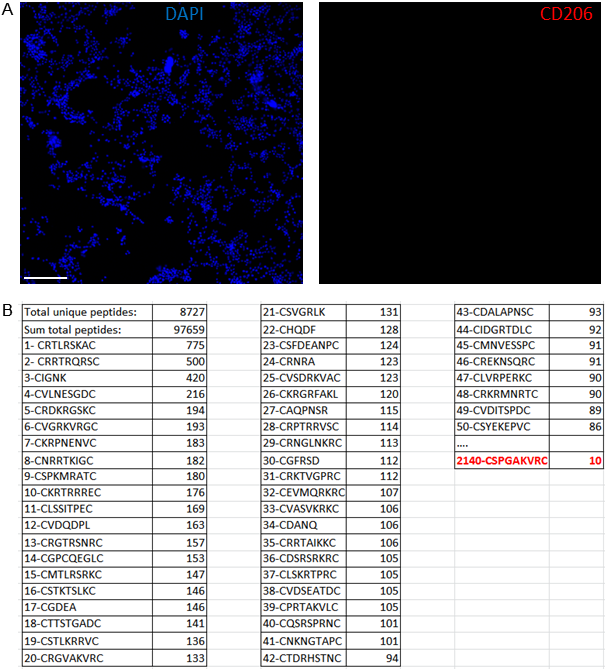


**Fig. S20. CSPGAKVRC is not selected in phage library screening on cultured CD206^-^ mouse macrophages.** **A**, Cultured RAW 267.4 mouse macrophages were fixed with PFA and stained with rat anti-CD206 (red) and counterstained with DAPI (blue). **B**, Phage display on RAW 267.4 cells. Cells were lifted using a cell scraper, centrifuged, brought to 4^o^C and incubated with 0.5mL of the same phage library used for the *in vivo* experiments (0.5mL of 7 x 10^10^ pfu/mL) + 0.5 mL of DMEM, at 4^o^C overnight. Cells were then washed four times resuspending in PBS and placing in a new tube every time, lastly the cells were suspended in LB + NP40%, lysed and phage insert were sequenced. The UNO clone is underrepresented in position 2140 of the list (red). Only the top 50 hits from the screen are shown. Scale bar: 50µm.


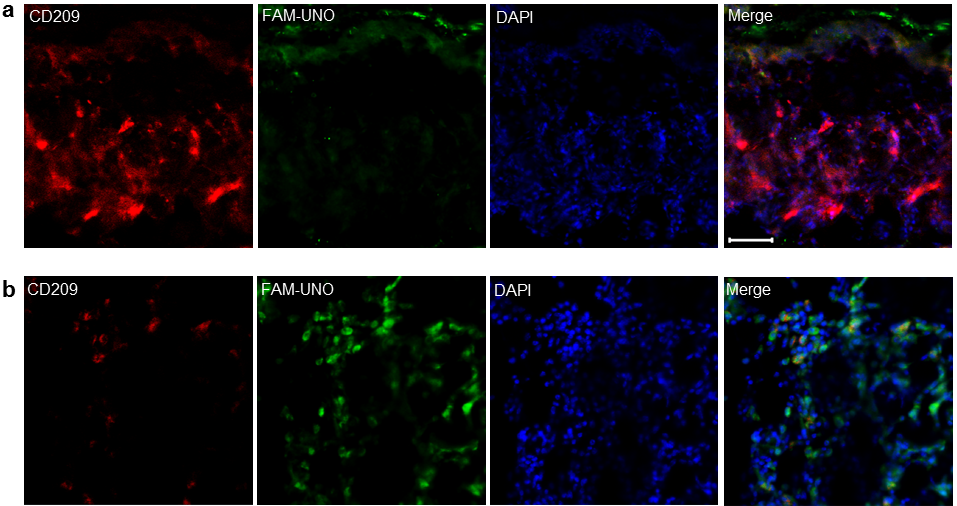


**Fig. S21. FAM-UNO does not bind to CD209. A,** Thirty nmoles of FAM-UNO were injected intravenously in healthy Balb/C mice. Peptide was allowed to circulate for 2 hours. Mice were then sacrificed and intestinal tissue was analyzed by immunofluorescence using rabbit anti-FAM (green) together with rat anti-CD209 (red) and counterstained with DAPI. **B,** Thirty nmoles of FAM-UNO were injected intravenously in mice bearing 4T1 tumors, 10 days after orthotopic inoculation of 10^6^ Cells. Peptide was let to circulate for two hours, mice were then sacked and tumor and tissues analyzed by immunofluorescence using rabbit Anti-FAM (green) together with rat anti-CD209 (red) and counterstained with DAPI (Blue). Images in A and B were taken using the same imaging conditions. Scale bar: 50µm (images in B are at the same scale as in A). Representative images from n=3 mice


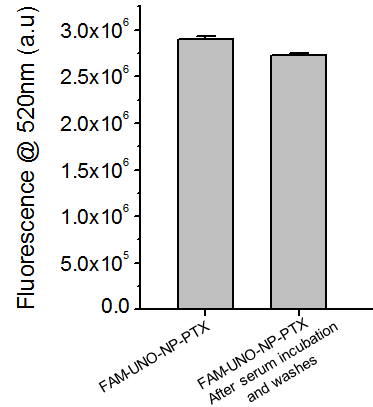


**Fig. S22. FAM-UNO does not significantly dissociate from nanoparticles after 6 hours of serum incubation.** 200µL of blood were extracted from the tail vein of a mouse bearing orthotopic 4T1 breast tumor (10 days after inoculation of 10^6^ cells) in a blood collection tube and plasma was separated by centrifugation. Later, 150µL of FAM-UNO-NP-PTX in PBS were mixed with 150µL of serum and incubated for 6 hours at 37 ˚C with shaking, then particles were washed by 3 centrifugation cycles (21000g for 30 minutes), and redispersed in 150µL of PBS to obtain “FAM-UNO-NP-PTX-Serum”. Then, 150µL of the original FAM-UNO-NP-PTX in PBS and 150µL of FAM-UNO-NP-PTX-Serum were placed in a 96 well plate and the fluorescence was measured using a FlexStation 3 Multi-Mode Microplate Reader (Molecular Devices) using 490nm excitation and collecting at 520nm. Mean + SEM from three independent experiments


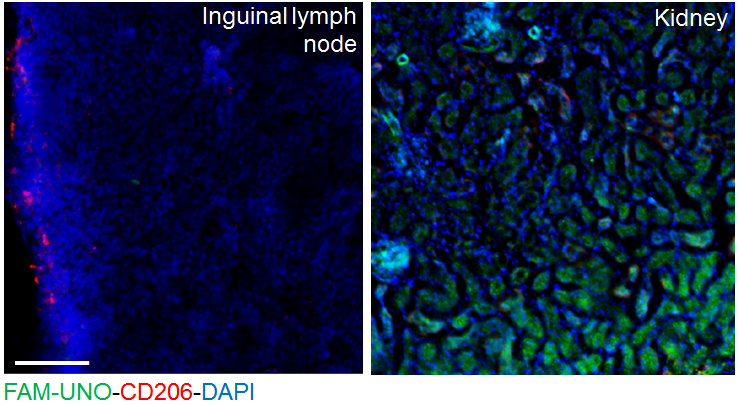


**Fig. S23. FAM-UNO does not home to healthy lymph nodes**. Thirty nmoles of FAM-UNO were injected intravenously in healthy Balb/C female mice. Peptide was allowed to circulate for 2 hours. Mice were then sacrificed, and inguinal lymph node and kidney analyzed by immunofluorescence using rabbit anti-FAM (green) together with rat anti-CD206 (red) and counterstained with DAPI (blue). Scale bars: 100µm. Representative images from n=3 mice.
